# Kinematic Biomarkers of Limb Shortening and Compensations in Hemiparetic Gait: A Systematic Review

**DOI:** 10.3390/s25154598

**Published:** 2025-07-25

**Authors:** Emmeline Montané, Lucille Lopez, Marino Scandella, David Gasq, Camille Cormier

**Affiliations:** 1 Department of Physical and Rehabilitation Medicine, University Hospital of Toulouse, 31300 Toulouse, France; montane.e@chu-toulouse.fr (E.M.); lopez.l@chu-toulouse.fr (L.L.); 2 Toulouse Neuroimaging Center (ToNIC), Inserm, Toulouse University, 31300 Toulouse, France; cormier.c@chu-toulouse.fr; 3 Gait Analysis Laboratory, Department of Pediatric Surgery, University Hospital of Toulouse, 31300 Toulouse, France; scandella.m@chu-toulouse.fr; 4 Motion Center Analysis, Department of Physiological Explorations, University Hospital of Toulouse, 31300 Toulouse, France

**Keywords:** gait analysis, hemiplegia, limb shortening, compensatory movements, toe clearance

## Abstract

**Highlights:**

**What are the main findings?**

- There is a lack of standardized and systematic descriptions for limb shortening and compensatory movements in hemiparetic gait.

**What is the implication of the main finding?**

- We proposed a set of relevant biomarkers to enhance standardization by systematically describing the deficit of shortening and the compensatory movements.

**Abstract:**

Background: Hemiparetic gait is characterized by reduced limb shortening during swing, increasing the risk of tripping and leading to compensatory strategies. Despite 3D gait analysis being the gold standard for gait assessment, there is no consensus on relevant kinematic biomarkers for limb shortening and compensatory movements. Methods: Systematic review querying five databases (PubMed, Cochrane, Scopus, PEDro, and Web of Science). We included articles that described at least one kinematic biomarker of the lower limb in the sagittal plane and at least one biomarker of the lower limb or pelvis in the transversal or frontal plane, or pelvis in the sagittal plane. Then, we collected kinematic biomarkers from these studies and identified those that seemed relevant to describe limb shortening and compensatory movements during the swing phase. Results: We included 40 studies and collected 385 biomarkers. Among them, 15 described limb shortening, 22 compensations, and 3 toe clearance. Analysis of 12 interventional studies showed that some biomarkers of shortening and compensation were more sensitive to change than others. Conclusions: This review highlights the lack of standardized description for limb shortening and compensatory movements in hemiparetic gait. A set of 13 relevant biomarkers is proposed to improve the interpretation of gait analysis and support consistent evaluation of therapeutic interventions.

## 1. Introduction

Hemiparesis is a motor disturbance caused by a central nervous system injury that usually leads to gait disorders [1]. Among post-stroke subjects, 64% achieve independent ambulation after 11 months of rehabilitation, while 14% still require assistance [2]. Hemiparetic subjects commonly exhibit abnormal gait patterns, including insufficient limb shortening during the swing phase of gait on the affected side [3,4], mainly due to muscle weakness and spastic overactivity [5]. Under physiological conditions, foot clearance during the swing phase of gait primarily results from limb shortening in the sagittal plane, enabled by the coordinated flexion of the hip, knee, and ankle joints [6]. Pelvic movements are minimal during the swing phase and mainly serve to limit the vertical displacement of the body’s center of mass, reducing energy expenditure [7]. In stroke survivors, the impaired ability to shorten the paretic limb in the sagittal plane often necessitates the use of compensatory movements in other planes of motion to ensure foot clearance and avoid tripping or falling [8]. However, these adaptations typically come at the cost of increased energy expenditure [9]. In the sagittal plane, compensatory strategies may include a steppage gait, characterized by exaggerated hip and knee flexion. In the frontal plane, hip hiking—elevation of the ipsilateral hip joint center—can facilitate swing phase clearance. In the transverse plane, circumduction and external rotation of the lower limb are commonly observed, primarily driven by hip abduction and external rotation. Compensatory strategies can also involve the contralateral limb, such as vaulting, where the stance leg performs plantarflexion during push-off to help the paretic limb clear the ground [10,11].

Improving limb shortening during the swing phase has been identified as a key therapeutic target for enhancing gait patterns of hemiparetic subjects, either by enhancing the recruitment of paretic flexor muscles or by weakening hypertonic extensor muscles. Various therapeutic strategies have been used, such as strengthening of the hip flexors and ankle dorsiflexors through rehabilitation programs [12], functional electrical stimulation (FES) of ankle dorsiflexors [13], botulinum toxin injections targeting knee extensors [14,15,16], orthotic devices such as ankle–foot orthoses (AFOs) [17,18,19], and neuro-orthopaedic surgery [20].

A comprehensive view of limb shortening is essential to evaluate the overall impact of these interventions on gait and to define relevant therapeutic goals. For instance, the FES of ankle dorsiflexors has been shown to have a detrimental effect on maximum knee flexion during swing [13]. However, synthesis studies rarely provide this broader kinematic perspective—either due to a lack of available data, as reported by some authors [20,21], or because they focus exclusively on a single joint level [17,18,19].

Moreover, evaluating limb shortening and compensatory movements simultaneously appears particularly valuable. The effect of botulinum toxin injections into the rectus femoris on improving knee flexion during swing—by approximately 5°—has been well documented [14,15,16]. However, their impact on compensatory movements has not been investigated. Yet reducing compensatory movements may help decrease the energy cost of walking and contribute to patients’ perceived improvement, just as much as improving limb shortening reduces the risk of tripping.

Three-dimensional instrumental gait analysis (3D-IGA) is considered the gold standard for investigating gait abnormalities, notably offering a standardized and detailed kinematic dataset that enables thorough evaluation of both limb shortening mechanisms and compensatory strategies. Its use is now recommended by the International Stroke Recovery and Rehabilitation Alliance for evaluating gait after stroke, particularly through global indices such as the Gait Deviation Index (GDI) and Gait Profile Score (GPS). These recommendations also emphasize the need for further work on biomechanical metrics—especially those aimed at identifying compensatory strategies—which is expected to be addressed in upcoming consensus efforts [22]. However, up to now, the selection of kinematic biomarkers to characterize gait abnormalities is left to the discretion of the investigator, which limits consistency across studies and clinical applications. Moreover, currently available systematic reviews on instrumental gait assessment in post-stroke individuals have not specifically focused on the swing phase or on compensatory movements associated with insufficient limb shortening [23,24,25]. This gap highlights the need for a more targeted and comprehensive synthesis of relevant biomarkers addressing these specific aspects of post-stroke gait impairment.

This systematic review aimed to establish how limb shortening and compensatory movements during the swing phase are currently conceptualized and measured in the existing literature. The secondary objective was to identify the most relevant 3D-IGA-derived, kinematic biomarkers for characterizing limb shortening and compensatory movements in hemiparetic subjects. Biomarker validity was assessed by reviewing the contexts in which they were used, and their sensitivity to change was analyzed through pre/post-intervention or with/without orthosis comparisons.

This investigation aimed to enhance practitioners’ understanding of the relationship between limb-shortening deficits and compensatory movements, potentially facilitating the evaluation of therapeutic interventions by assessing reductions in compensatory movements alongside improvements in limb shortening.

## 2. Materials and Methods

This systematic review followed the Preferred Reporting Items for Systematic Reviews and Meta-Analyses (PRISMA) statement and used a predefined research protocol.

### 2.1. Search Strategy

A comprehensive search was conducted on 31 March 2023 across MEDLINE, COCHRANE, Web of Science, PEDro, and Scopus using MeSH and non-MeSH terms, including hemiplegia, stroke, brain injury, gait, and kinematics. Detailed search strategies for all the databases are available in Appendix A. No filters, date restrictions, or additional searches through cited references were applied.

### 2.2. Eligibility Criteria

The study inclusion criteria were as follows: adults (>18) with hemiparesis due to central nervous system disease, who underwent a 3D gait analysis using an optoelectronic system either on a treadmill or overground. At least one kinematic data point for limb shortening and compensatory movements was required. Exclusion criteria were non-gold-standard systems, non-hemiplegic conditions, children, animals, and spinal injuries. Robot-assisted studies were included if the system was used for rehabilitation without assistance during gait analysis. Eligible studies were published in English, Spanish, German, or French and included observational, interventional, and case series designs.

### 2.3. Study Selection Process

Two independent reviewers (EM and LL) used the Rayyan online tool (https://www.rayyan.ai, accessed on 31 March 2023) for duplicate removal and screening of titles and abstracts. Full texts were reviewed for eligibility, and disagreements were resolved through consensus. A third reviewer (DG) was consulted if necessary.

### 2.4. Data Collection Process and Data Items

Two independent reviewers (EM and LL) extracted data, without contacting authors for missing information. Collected data included: the name of the first author, year of publication, study design, study objective, number of subjects, population characteristics (mean age, disease, time since stroke for post-stroke subjects), inclusion and exclusion criteria, system analysis, kinematic model, experimental conditions (e.g., treadmill or overground walking), intervention characteristics and outcome measures.

For interventional studies, intervention characteristics were extracted from those describing a pre-/post-protocol or a with/without orthotic device comparison. When available, information on protocol duration, frequency, and therapeutic target (e.g., muscle, joint, or movement strategy) was recorded.

Outcome measures included all quantitative kinematic biomarkers described in the study. Only biomarkers derived from three-dimensional optoelectronic motion capture systems were retained. Spatiotemporal, kinetic, or EMG parameters were not considered.

Kinematic biomarkers were categorized into: (1) limb-shortening biomarkers (hip, knee, ankle in the sagittal plane); and (2) compensatory movements biomarkers (hip, knee, ankle and pelvic movements in the frontal and transversal planes as well as pelvic movements in the sagittal plane). Toe clearance biomarkers were also collected as a result of both limb shortening and compensations. Biomarkers were identified by direction of movement, quantitative description, and gait cycle timing. Only swing-phase-specific biomarkers with quantitative data were included. Biomarkers lacking these criteria or not describing limb shortening or compensatory movements were excluded. Finally, we compiled a list of relevant kinematic biomarkers for describing limb shortening (e.g., hip flexion, knee flexion, ankle dorsiflexion, toe clearance, and global limb shortening) or compensatory movements (e.g., hip hiking, circumduction, posterior pelvic tilt, posterior pelvic rotation, and vaulting). Studies reporting at least one limb shortening and one compensatory movement biomarker were selected for qualitative analysis.

To complement the descriptive synthesis, we conducted a secondary quantitative synthesis. For this purpose, we identified a subset of interventional studies that included pre- and post-intervention data or with/without orthosis comparisons. From these studies, relevant swing-phase-specific kinematic biomarkers were extracted with their associated numerical values, when reported, to assess the sensitivity of these biomarkers to intervention.

### 2.5. Study Risk of Bias Assessment

For the qualitative analysis, risk of bias was deemed irrelevant as the focus was on biomarkers rather than study outcomes. Consequently, no formal bias assessment was conducted. For the quantitative analysis, two independent reviewers (EM and LL) evaluated methodological quality using JBI’s critical appraisal tools.

### 2.6. Synthesis Methods

A descriptive synthesis summarized study characteristics and biomarker usage. A Circos plot was created using R (version 4.3.2, Circlize package 0.4.16) [26,27] to visually represent the distribution and frequency of biomarker usage. In this plot, each study was linked to the biomarkers it described, allowing for immediate visual identification of (1) which biomarkers were most frequently reported, and (2) the heterogeneity in marker selection across studies.

### 2.7. Effect Size

To assess whether interventions or orthotic devices increased limb shortening while reducing compensatory movements, effect sizes were calculated. If necessary, standard deviation was derived from standard error based on sample size. For paired pre/post-intervention comparisons, Hedges’ g with 95% confidence intervals was used. Confidence intervals excluding zero were considered statistically significant [28]. Effect sizes were classified as small (0.2), moderate (0.2–0.8), or large (>0.8) [29].

## 3. Results

### 3.1. Study Selection

**Figure 1:**
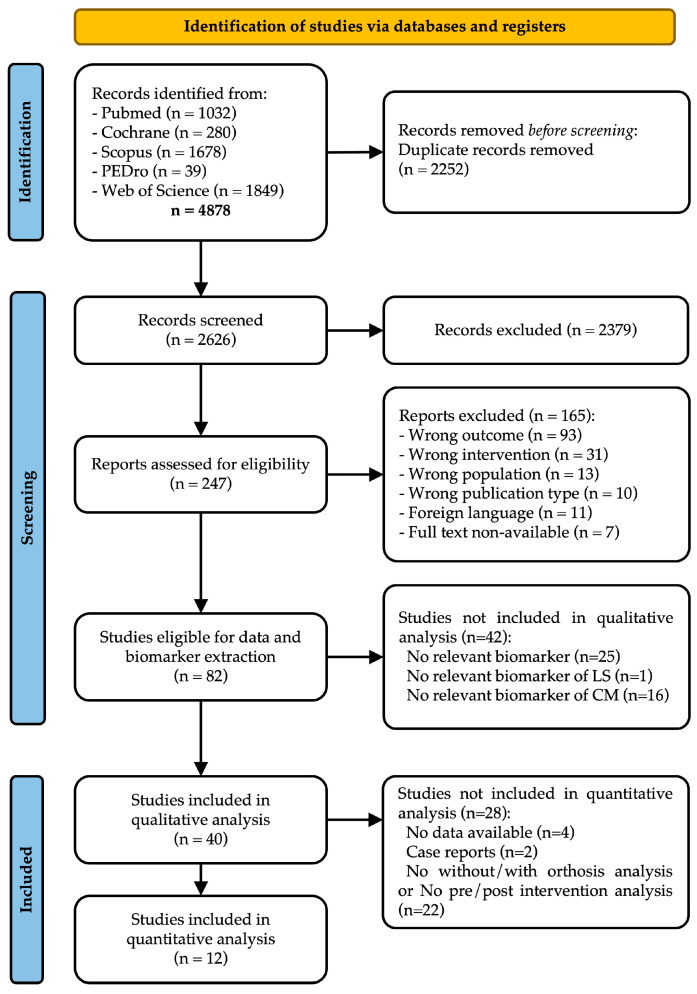
PRISMA flowchart of studies’ selection process.

**Figure 2:**
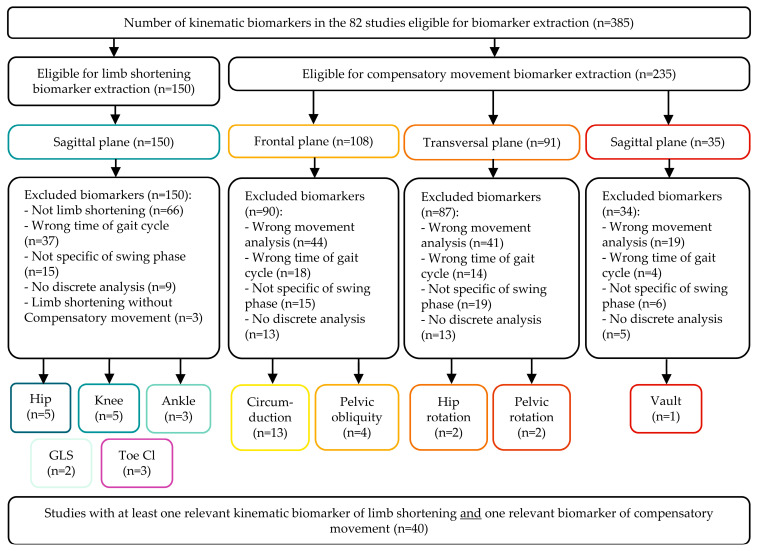
Flow chart of biomarkers’ selection process. Biomarkers related to limb shortening are outlined in blue-green, those reflecting compensatory movements in red-orange, and those associated with toe clearance in purple.

The selection process is presented in the PRISMA flowchart (Figure 1). A database search identified 4878 records. 2626 unique records were identified since this value excluded the duplicates removed, from which 82 studies met the inclusion criteria after abstract and full-text screening. The main reasons for exclusion were the absence of frontal/transversal plane data, sagittal pelvic analysis, or optoelectronic system use. Biomarker extraction followed (Figure 2), identifying 385 biomarkers: 15 for describing limb shortening, 24 for compensatory movements, and 3 for clearance. Irrelevant biomarkers and exclusion reasons are listed in Supplemental Digital Content Tables S1 and S2. Exclusions mainly resulted from incorrect gait cycle timing (*n* = 37, stance phase data) and inappropriate compensatory movement analysis (*n* = 104, lacking directional data). Following biomarker selection, 40 of the 82 eligible studies were included in the qualitative analysis. Characteristics and reasons for excluding 42 studies are detailed in Supplemental Digital Content Table S3. No additional records were found in manual reference searches.

### 3.2. Population and Studies Characteristics

Among the 40 studies included, 22 were observational and 18 were interventional. A total of 891 participants were analyzed, with a mean (SD) of 22.3 (19.8) participants per study. The mean (SD) age across studies was 54.8 (7.6) years. Of these participants, 878 were post-stroke individuals, 8 had cerebral palsy, and 5 had traumatic brain injury. Within the stroke population, 616 participants (70.2%) were evaluated in the chronic phase, 182 (20.7%) in the subacute phase, 31 (3.5%) in mixed subacute-chronic cohorts [30], and the time since stroke was not specified for 49 participants (5.6%) [31,32,33].

Most studies (*n* = 31) were conducted in motion analysis laboratories, while the remaining nine used treadmill-based assessments. A total of 33 studies employed optoelectronic motion capture systems. Among these, the most commonly used system was Vicon (*n* = 22), followed by Motion Analysis (*n* = 7). Other systems included Qualisys, Dvideo, BTS, SMART-D140, Mac 3D, Oqus, and PhaseSpace, each used in one or two studies.

Kinematic modeling approaches varied considerably across studies. Custom-built models focusing solely on the pelvis and lower limbs were used in eight studies, while four studies employed custom models including the trunk, and one study included both trunk and head. Full-body custom models were reported in four studies. Standardized models were frequently used, including Plug-In Gait (*n* = 12), Helen Hayes or its modified versions (*n* = 5), and the Modified Cleveland Clinic model (*n* = 3). Less commonly used models included SAFLo, Leardini, and Total3Dgait (one study each). One study did not specify the model used.

Among the interventional studies, eight investigated kinematic parameters with and without assistive devices: mainly AFOs [19,34,35,36,37,38,39], but also a powered knee orthosis [40] and a soft wearable robot [41]. Five studies assessed changes in kinematics before and after different types of rehabilitation programs, including: functional electrical stimulation [33], rhythmic auditory stimulation [42], treadmill rehabilitation with cross-tilt [43], transcranial direct current stimulation combined with treadmill training [44], and photobiomodulation therapy combined with a static magnetic field [45]. The included studies’ characteristics are summarized in Table 1.

Prior to biomarker exclusion, the studies highlighted a median (min–max) of 11 (2–91) biomarkers, including 7 (1–32) limb-shortening biomarkers and 3 (1–59) compensatory biomarkers.

Following biomarker exclusion, the studies highlighted a median (min–max) of 5 (2–10) biomarkers, including 2 (1–6) limb-shortening biomarkers and 2 (1–7) compensatory biomarkers.

### 3.3. Selected Biomarkers

#### 3.3.1. Limb Shortening

**Figure 3:**
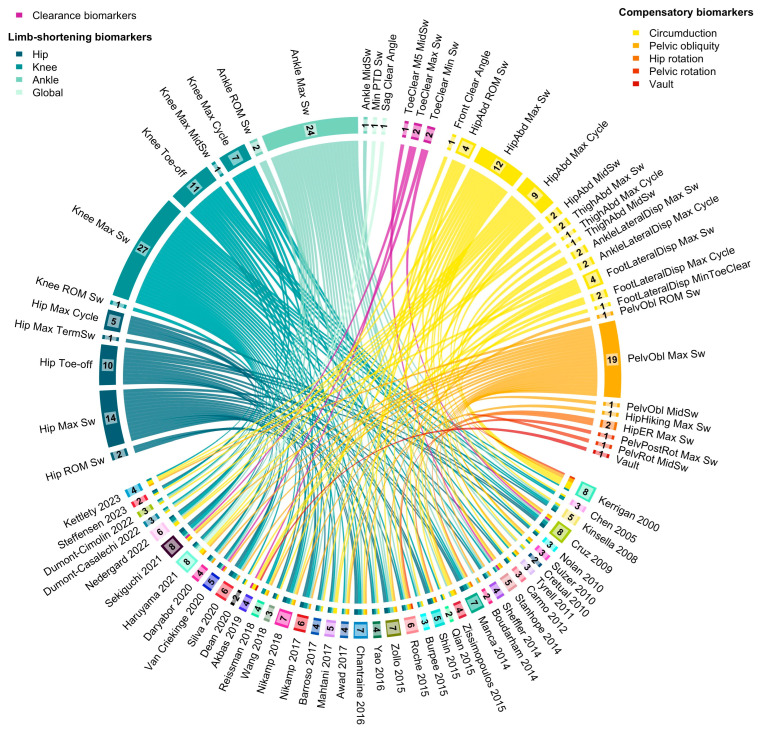
Circos-plot linking the included studies with their relevant kinematic biomarkers for limb shortening and compensatory movement description. Biomarkers are displayed in the upper part of the figure: limb-shortening biomarkers are shown on the left in blue-green, compensatory movement biomarkers on the right in yellow-orange, and clearance-related biomarkers in the centre in purple. The studies using these biomarkers are listed in the lower part of the figure. The number of relevant biomarkers per study and the number of times each biomarker was used are reported in black for each study or biomarker, respectively. The list of biomarker abbreviations is available in Table 2.

We identified 15 kinematic biomarkers as relevant for describing limb shortening during the swing phase. These included five hip flexion biomarkers—maximum hip flexion during swing, hip flexion at toe-off, maximum flexion at terminal swing, maximum hip flexion during the gait cycle, and hip flexion range of motion during swing. Similarly, five knee flexion biomarkers were reported, including maximum knee flexion during swing, knee flexion at toe-off, knee flexion at mid-swing, maximum knee flexion during the cycle, and knee flexion range of motion during swing. Three ankle dorsiflexion biomarkers were included: maximum dorsiflexion during swing, ankle angle at mid-swing, and ankle dorsiflexion range of motion during swing. Additionally, two biomarkers assessed global limb shortening: minimal pelvic–toe distance and sagittal clearance angle (Figure 3).

Among these, maximum knee flexion during swing was the most frequently used biomarker (reported in 27 studies) [10,19,30,32,34,37,38,39,40,41,43,44,45,46,47,48,52,53,54,55,56,57,58,62,64,65,67], followed by maximum ankle dorsiflexion during swing (24 studies) [10,19,30,31,32,34,35,37,38,39,41,44,45,48,52,53,54,55,56,57,58,59,64,65], and maximum hip flexion during swing (14 studies) [10,19,32,34,37,38,39,53,55,56,57,58,64,65]. Other joint-specific biomarkers, such as hip flexion at toe-off (10 studies) [32,34,38,39,42,48,49,53,57,65] or knee flexion at toe-off (11 studies) [32,34,38,39,43,47,48,49,53,57,65], were less consistently used. Composite or segmental measures like the minimal pelvic–toe distance and sagittal clearance angle were [14,25,27,29,32,33,34,35,36,38,39,40,41,42,43,47,48,49,50,51,52,53,54,58,60,61,63] reported in only one study [47].

Moreover, temporal parameters—such as toe-off (11 studies) [32,34,38,39,43,47,48,49,53,57,65], mid-swing (4 studies) [36,42,46,52], or terminal swing (1 study) [42]—were not consistently specified across studies. Range-of-motion biomarkers (e.g., hip or ankle ROM during swing) were rarely used (1 study) [37].

#### 3.3.2. Toe Clearance

Five studies reported toe clearance using five different biomarkers (Figure 3) [34,52,61,63,65]. These included the vertical displacement of the fifth metatarsal head at mid-swing, the maximal vertical displacement of the toe marker during swing, and its minimal vertical displacement, which were used in one or two studies each. Only one study reported sagittal and frontal clearance angles, both aiming to reflect the combined contribution of limb shortening and compensatory movements to overall limb clearance.

#### 3.3.3. Compensatory Movements

Among the 22 kinematic biomarkers relevant for compensatory movements (Figure 3), 13 quantified circumduction and 4 quantified hip hiking. Circumduction was the most frequently reported compensatory movement (13 biomarkers, 32 studies), assessed via maximum hip abduction during swing (12 studies) [30,31,34,39,40,43,48,52,53,55,57,59] or maximum hip abduction during cycle (9 studies) [30,44,45,46,51,58,60,66,67]. It was also quantified using lateral paretic limb displacement in the frontal plane with two biomarkers based on ankle or heel marker (4 studies) [36,50,58,62] and three biomarkers based on foot or toe marker (6 studies) [33,41,47,54,61,67]. Three studies proposed quantifying circumduction by measuring thigh abduction angle [46,64,65] instead of hip abduction. Hip hiking was the second most reported movement (4 biomarkers, 21 studies), mainly via maximum pelvic obliquity during swing [10,19,32,34,35,36,38,39,40,43,46,50,52,55,57,63,64,65,67], with one study measuring hip marker elevation in centimeters in the frontal plane [41].

Posterior pelvic rotation [46,55] and hip external rotation [42,53] were poorly described. Vaulting was quantified in one study [61] by measuring the maximum of non-paretic ankle dorsiflexion during stance. Posterior pelvic tilt was not considered a compensatory movement, as it primarily facilitates forward limb progression rather than compensating for limb shortening.

### 3.4. Quantitative Analysis

The JBI critical appraisal tools were used to assess risk of bias (Supplemental Digital Content Table S4). The primary concern in quasi-experimental studies was the absence of a control group, while in RCTs, the main issue was non-concealed treatment allocation. However, such biases did not provide sufficient grounds to exclude studies since our primary objective was to analyze 3D-IGA biomarkers, which were not influenced by study bias.

A 12-study quantitative analysis assessed effect sizes for therapeutic interventions (pre- and post-intervention and with/without orthosis) on kinematic biomarkers to evaluate which were the most sensitive to change (Table 3; full effect sizes in Supplemental Digital Content Table S5). Studies are listed in Table 1.

Across the 101 limb-shortening biomarkers analyses, moderate to large effect size were observed for: maximum ankle dorsiflexion increase (20/27 analyses, nine are significant) [34,35,37,38,39,41,44,45,58], maximum knee flexion increase (9/27 analyses, three are significant) [39,42,44,58], maximum knee flexion decrease (3/27, one is significant) [34,37], and hip flexion at toe-off decrease (5/11 analyses, three are significant) [39].

Across the 47 compensatory movement biomarkers analyses, intervention effects on circumduction biomarkers were heterogeneous. Positive moderate to large effect sizes were observed for: maximum hip abduction during the cycle (8/15 analyses, two are significant) [44,45,58], maximum lateral displacement of the ankle during the cycle (1/3 analysis, significant) [58]. Negative moderate to large effect sizes were observed for: maximum hip abduction during swing (1/9 analyses, significant) [39], maximum hip abduction during the cycle (1/15 analyses, significant) [60], and maximal lateral displacement of the foot during the cycle also decreased (1/1 analysis, significant) [33]. Pelvic obliquity and hip hiking biomarkers had poor to moderate effect sizes.

## 4. Discussion

This systematic review analysed the use of 3D-IGA biomarkers to assess limb shortening and compensatory movements specifically during the swing phase of hemiplegic gait. The objective was to evaluate how these phenomena are defined in the literature and identify the most relevant kinematic biomarkers for their quantification. The review highlighted a critical gap: only 40 out of 2626 studies (1.52%) measured both limb shortening and compensatory movements concurrently, revealing a lack of comprehensive descriptions and standardization in kinematic biomarkers. Although 3D-IGA protocols adhere to standardization, the selection and interpretation of biomarkers vary according to clinician preference, contributing to significant heterogeneity in kinematic data.

### 4.1. Respective Contributions of Joints on Limb Shortening

A precise description of limb shortening in hemiparetic gait requires a detailed kinematic analysis focused on the swing phase. However, many studies did not emphasize this phase, often lacking quantitative biomarkers (Supplemental Digital Content Table S3).

Definitions of mid-swing varied considerably; some identified it as the midpoint of the swing phase [46], aligning with minimal toe clearance in healthy subjects [6], while others defined it based on thigh advancement [7]. Notably, studies suggest that minimal toe clearance in hemiparetic gait may occur earlier than in healthy individuals, making it an unreliable indicator of limb shortening [68,69]. Instead, mid-swing can be defined as the moment when the toe marker aligns beneath the hip marker, corresponding to maximum limb advancement and offering a reliable reference for assessing both limb shortening and compensatory movements [6,64]. This alignment ensures that limb clearance is at its most critical phase, making it an optimal point for evaluation [68].

Selecting hip and knee biomarkers during pre-swing is relevant, as they predict flexion movements in swing. This phase influences maximum knee flexion, with muscle forces at pre-swing determining knee flexion velocity at toe-off, which strongly correlates with maximum knee flexion during swing [70].

Accordingly, and in line with the literature, we selected biomarkers that describe limb shortening at maximum flexion for the hip, knee, and ankle simultaneously to identify its origin. Additionally, we included biomarkers assessing limb shortening at maximum global limb shortening or when the vertical projection of the toe marker aligns with the hip marker. Although mid-swing biomarkers were included, they are not recommended due to variability in their concordance with limb shortening in hemiparetic gait [68].

Most studies assessed limb shortening via segmental kinematic angular analysis, quantifying the decrease in hip, knee, and ankle flexion during the swing phase (Figure 3). While informative, this approach does not fully capture the contribution of each joint to global limb shortening. An alternative method proposed by Prado-Medeiros et al. [71] involves measuring elevation angles of the thigh, shank, and foot relative to a vertical reference line, eliminating dependency on above and below-segment positioning. These relevant biomarkers are not illustrated in Figure 3 because the study did not describe any compensatory movement. Another interesting approach is to measure in millimeters both global limb shortening and the shortening of the hip–toe distance and the component of this shortening due to the hip, knee, and ankle [68].

### 4.2. Toe Clearance and Global Limb Shortening

Toe clearance, representing foot elevation during swing, was inconsistently measured across studies, with only five reporting on this parameter using three different biomarkers. Toe clearance could be estimated from the vertical displacement of the second or the fifth metatarsal head marker, depending on the kinematic model. The height during swing was corrected by the height during stance [34,68]. The relevance of this parameter for assessing post-stroke intervention efficacy is questionable, as it appears to be insensitive to change. For instance, Cruz et al. demonstrated an improvement in limb shortening by wearing an AFO, resulting in increased maximum ankle dorsiflexion during swing and decreased compensatory movements such as hip hiking. However, the intervention did not improve toe clearance [34]. This lack of improvement could be attributed to minimal toe clearance, which may not exceed measurement error or represent global limb clearance. Thus, improvements in limb shortening or compensations do not necessarily correlate with changes in toe clearance, although patients may experience improved comfort from reduced compensations. Quantifying maximal toe clearance may not be relevant, as it occurs late in the swing phase and does not reflect limb shortening in the critical part of the swing.

Haruyama et al. proposed minimum pelvic–toe distance as a measure of global limb shortening, defined as the linear distance between the anterior superior iliac spine and a toe marker [64]. This measure accounts for hip flexion, knee flexion, ankle dorsiflexion, and anterior pelvic tilt. This biomarker correlated strongly with gait quality, speed, and maximum knee flexion, remaining unaffected by frontal plane compensations, probably due to the omission of hip hiking in this index. A suitable biomarker for measuring global limb shortening would be limb length from hip to toe, eliminating pelvic tilt effects on apparent shortening.

Additionally, Haruyama et al. introduced the Sagittal Clearance angle, summing hip, knee, and ankle flexion, and the Frontal Clearance angle, summing hip hiking and circumduction [64]. The maximal Sagittal Clearance angle correlated positively with global limb shortening, while the Frontal Clearance angle showed a negative correlation. Although the term “clearance” may seem confusing, the distinction highlights the respective contributions to functional limb shortening. Assessing these biomarkers post-intervention could provide valuable insights.

### 4.3. Compensatory Movements

Circumduction was the most frequently quantified compensatory movement, with 12 biomarkers identified. Maximum abduction during the gait cycle was commonly measured, though its role in limb shortening is debated [64]. Some studies measured lateral heel displacement, while others used hip abduction angles, both of which were influenced by pelvic motion and hip hiking (Figure 3). Kerrigan et al. proposed quantifying circumduction via the coronal thigh angle relative to a vertical line, eliminating pelvic influence and allowing independent measurement [46].

Hip hiking was consistently defined using pelvic obliquity angles, with most studies assessing maximum obliquity during swing. However, obliquity during the entire gait cycle is less relevant due to its physiological role in stance. Only one study proposed quantifying hip elevation in centimeters [41]. Our review revealed that a clear definition of hip hiking and circumduction is still lacking, thus emphasizing the need for independent quantification. It seems especially pertinent to describe thigh abduction [46] and pelvic elevation [41] in conjunction.

Posterior pelvic rotation and hip external rotation were rarely examined. Additionally, pelvic motion biomarkers in the transversal and sagittal planes lacked directional information, complicating the determination of compensation direction. Measures of pelvic range of motion were often irrelevant, as they covered the entire gait cycle rather than focusing on swing-phase compensations.

For pelvic compensations, it is crucial to select biomarkers specific to the swing phase to distinguish them from other compensations, to describe them quantitatively, and provide directional information.

A promising approach by Pongpipatpaiboon et al. [68] described compensatory movements and their components (e.g., paretic side pelvic obliquity, non-paretic hip elevation, and foot elevation due to circumduction) in millimeters, facilitating direct comparison of shortening and compensatory changes accounting for proportional changes in each joint. The change in dorsiflexion in millimeters can be compared to the change in pelvic obliquity in millimeters. Although this study was excluded due to non-optoelectronic methodology, its approach remains highly relevant.

### 4.4. Quantitative Analysis

The quantitative analysis identified biomarkers with a moderate to large effect size following intervention. A meta-analysis was unfeasible due to heterogeneity in interventions, biomarkers and populations.

Among the 12 studies, biomarkers for limb shortening and compensatory movements showed varying degrees of responsiveness to intervention. Three limb-shortening biomarkers showed significant effect sizes post-intervention. Maximum ankle dorsiflexion during swing demonstrated the highest sensitivity, with moderate to large effect sizes in 20 of 27 analyses (9 statistically significant). Maximum knee flexion exhibited mixed effects, increasing in pre- and post-intervention studies but decreasing in with/without orthosis studies, suggesting that AFOs did not increase knee flexion or may even worsen it due to altered propulsion [72]. Hip flexion at toe-off, a key determinant of the maximum knee flexion during swing [73], showed significant responsiveness in five analyses.

For compensatory movements, circumduction biomarkers displayed substantial effect sizes post-intervention, though the direction varied. Some interventions reduced maximum hip abduction during swing or during cycle and lateral foot displacement during cycle, aligning with post-intervention or orthosis goals to minimize compensatory movements. Positive effect sizes were found for maximum hip abduction during swing and ankle lateral displacement during the cycle. All significant results came from one study on intensive stepping training in post-stroke patients, where increased frontal plane strategies likely resulted from higher walking speed or compensatory adaptation [58]. Although the direction of effect sizes varied, circumduction biomarkers were sensitive to changes due to interventions.

Conversely, pelvic obliquity and hip hiking biomarkers were largely insensitive to intervention-induced changes, potentially due to measurement variability in 3D gait analysis, where frontal plane errors can reach 2° [74].

These findings highlight limb shortening and compensatory biomarkers as sensitive to change and valuable for assessing intervention efficacy in gait analysis, reinforcing their importance in future studies.

### 4.5. Limitations

This study’s primary limitation was the heterogeneity of included studies, preventing a meta-analysis of intervention effects on kinematic biomarkers. Variability in study objectives also led to differences in extracted biomarkers, as some studies did not explicitly focus on limb shortening or compensatory movements. Furthermore, restricting the review to studies analyzing both limb shortening and compensatory movements may have excluded valuable research examining only one aspect.

Additionally, this review focused exclusively on 3D-IGA using optoelectronic systems, the gold standard for gait analysis. However, alternative gait analysis methods, more commonly used in clinical practice, were not included, potentially limiting the generalizability of findings. Differences in kinematic models likely influenced biomarker availability across studies. While technological advances may have facilitated access to full-body models, we observed no clear temporal trend—possibly because research teams tend to reuse familiar models for consistency and comparability. Nonetheless, all models used were sufficient to capture key features of limb shortening and compensatory strategies. Thus, improvements over time may reflect a growing awareness and evolving analytical focus rather than advances in modeling alone.

### 4.6. Perspectives

The absence of a standard definition for limb shortening and compensation in 3D-IGA complicates their role in global limb shortening. Akbas et al. suggest that circumduction may result from abnormal muscle coordination rather than a compensatory strategy [61]. We hypothesize that improving foot-floor clearance via limb shortening interventions (e.g., orthotics, rehabilitation, botulinum toxin) could reduce compensatory movements and energy expenditure during gait in hemiplegic individuals. Quantitative analysis suggests that both limb shortening and compensatory movement biomarkers could be useful in evaluating clinical practices. However, kinematic data variability prevents definitive conclusions, and meta-analysis was unfeasible.

Standardizing kinematic data measurement in 3D-IGA is crucial to understanding limb shortening, compensations, and gait quality. We propose key biomarkers in Table 4 to guide future studies, facilitating standardization and potential meta-analysis. We did not propose a toe clearance biomarker, as existing ones inadequately reflect tripping risk, capturing only combined limb shortening and compensatory effects. Future research should identify a more precise biomarker that better represents the subjective experience of tripping.

## 5. Conclusions

In conclusion, this systematic review identified biomarkers currently used in 3D-IGA to quantify limb shortening and compensatory movements. The lack of standardization in data extraction hinders the understanding of their relationship and impact on gait patterns. To address these gaps, we recommend a core set of biomarkers for defining global limb shortening, circumduction, and hip hiking, aiming to improve data standardization and enhance gait analysis in hemiplegic patients.

## Figures and Tables

**Table 1:** Studies characteristics.

Author	Design	Intervention	Population	Number of Patients	MOCAP	Main Objective
Kerrigan, 2000 [46]	Observational study	/	Chronic stroke	23	Laboratory. Optoelectronic. Vicon. SAFLo model	To define and propose clinically useful quantitative measurements of hip hiking and circumduction using a standard three-dimensional motion analysis technique.
Chen, 2005 [47]	Observational study	/	Chronic stroke	6	Laboratory. Optoelectronic. Vicon. Custom model including trunk.	To compare the gait of individuals with post-stroke hemiparesis and non-disabled controls while walking on a treadmill at matched speeds.
Kinsella, 2008 [48]	Observational study	/	Chronic stroke	23	Laboratory. Optoelectronic. Vicon. Plug In Gait model	To determine if the gait patterns of stroke participants with equinus deformity of the foot could be categorised into homogeneous subgroups using temporal distance and joint kinematic and kinetic measures. If this is the case, detail the differences in temporal distance and joint kinematics and kinetic measures between these groups.
Cruz, 2009 [34] *	Interventional study: pilot	With/without AFO	Chronic stroke	9	Laboratory. Optoelectronic. Motion analysis. Custom model (pelvis and lower limbs)	To quantify adaptive changes in the three-dimensional kinematics of the paretic lower limb and pelvis.
Nolan, 2010 [49]	Observational study: case report	/	Chronic stroke	1	Laboratory. Optoelectronic. Vicon. Plug In Gait model	To evaluate the effect of a dynamic AFO on ambulatory function during walking in one individual with post-stroke hemiplegia.
Sulzer, 2010 [40] **	Interventional study	Walk with/without the assistance of a powered knee orthosis	Chronic stroke	9	Treadmill. Optoelectronic. Vicon. Custom model (pelvis and lower limbs)	To identify whether abnormal frontal plane behaviours are in response to local knee impairment or a manifestation of an abnormal multisegmental motor programme.
Cretual, 2010 [31]	Observational study	/	Hemiplegic (unspecified)	13	Laboratory. Optoelectronic. Vicon. Custom model (pelvis and lower limbs)	This article describes a new multivariate measure of overall gait pathology called the Gait Deviation Index (GDI).
Tyrell, 2011 [50]	Observational study	/	Chronic stroke	20	Treadmill. Optoelectronic. Vicon. Modified Cleveland Clinic model	To investigate how speed affects not only spatiotemporal gait deficits but also sagittal- and frontal-plane gait kinematics.
Carmo, 2012 [51]	Observational study	/	Stroke	14	Laboratory. Optoelectronic. Dvideo. Custom full-body model	To analyse the kinematics of arm and leg movements during the stroke gait.
Stanhope, 2014 [52]	Observational study	/	Chronic stroke	21	Treadmill. Infrared. Motion Analysis. Custom model including trunk.	To investigate the relationship between self-selected walking speed and the kinematic patterns related to paretic foot clearance during post-stroke walking.
Sheffler, 2014 [10] **	Interventional study: secondary analysis of RCT	/	Chronic stroke	108	Laboratory. Optoelectronic. Vicon. Plug In Gait model	To evaluate, using quantitative gait analysis, the relationship between BMI and spatiotemporal, kinematic, and kinetic gait parameters in individuals with chronic hemiparesis.
Boudarham, 2014 [35] *	Interventional study	With/without DAFO	Chronic stroke	12	Laboratory. Optoelectronic. Motion Analysis. Helen Hayes model	To evaluate the effectiveness of an elastic DAFO on gait in hemiplegic patients with foot equinus due to spasticity of the triceps surae and to quantify the biomechanical adaptations induced by the DAFO on the paretic lower limb.
Manca, 2014 [53]	Observational study	/	Chronic stroke	49	Laboratory. Optoelectronic. Vicon. Total3Dgait model	To focus on the role of foot–ankle complex dysfunction in gait patterns in hemiplegic patients using a gait analysis protocol that allows full assessment of ankle–foot complex kinematics in the three planes of space.
Zissimopoulos, 2015 [36] **	Interventional study	With/without AFO	Chronic stroke	13	Laboratory. Optoelectronic. Helen Hayes model	To investigate whether an AFO improves ML foot-placement ability during ambulation in individuals with post-stroke hemiplegia.
Qian, 2015 [33] *	Interventional study	Before/after training with FES on tibialis anterior and rectus femoris	7 strokes, 1 TBI	8	Laboratory. Optoelectronic. Motion Analysis. Custom model including trunk.	To design and implement a multichannel dynamic functional electrical stimulation system and investigate acute effects of functional electrical stimulation of the tibialis anterior and rectus femoris on ankle and knee sagittal-plane kinematics and related muscle forces of hemiplegic gait.
Shin, 2015 [42] *	Interventional study	Before/after gait training with rhythmic auditory system	11 strokes, 7 cerebral palsy	18	Laboratory. Optoelectronic. Vicon. Plug In Gait model	To refine the effects of gait training with RAS in order to confirm the changes in both kinematic and temporospatial characteristics in patients with hemiplegia.
Burpee, 2015 [54]	Observational study	/	Chronic stroke	26	Laboratory. Optoelectronic. Vicon. Custom model (pelvis and lower limbs)	To determine the spatiotemporal, kinematic, and kinetic characteristics of the paretic lower extremity associated with naturally occurring unsuccessful foot clearance in participants with chronic hemiparesis secondary to stroke.
Roche, 2015 [55]	Observational study	/	Chronic stroke	60	Laboratory. Optoelectronic. Motion analysis. Helen Hayes model	To evaluate the relationship between: (i) peak ankle dorsiflexion and peak hip flexion during the swing phase of the gait cycle in stroke patients using a biomechanical approach (3D-IGA); (ii) the maximal voluntary strength of the hip and ankle dorsiflexor muscles evaluated clinically and the respective peak hip flexion and peak ankle dorsiflexion in swing; (iii) the spasticity of ankle plantar flexor muscles and ankle kinematics in the sagittal plane during swing.
Zollo, 2015 [37] *	Interventional study: crossover	3 walk conditions: without AFO/with dynamic AFO/with solid AFO	Chronic stroke	10	Laboratory. Infrared. BTS Smart System. Plug In Gait model	Comparative evaluation of two commercial AFOs with different mechanical properties (solid vs. dynamic AFO) by means of quantitative indicators of subject gait capabilities
Yao, 2016 [56] **	Interventional study: pilot	/	Chronic stroke	4	Laboratory. Optoelectronic. Vicon. Plug In Gait model	To obtain an initial insight into kinematic and kinetic walking patterns resulting from an implanted FES system in patients with drop foot due to stroke.
Chantraine, 2016 [57]	Observational study	/	Chronic stroke	26	Laboratory. Optoelectronic. Qualisys System. Leardini model	To propose a gait classification system for adult patients with hemiparesis.
Awad, 2017 [41] *	Interventional study	With/without soft wearable robot	Chronic stroke	8	Treadmill. Infrared. Vicon. Custom model (pelvis and lower limbs)	To investigate the effects of exosuit assistance on common post-stroke gait impairments and compensations.
Mahtani, 2017 [58] *	Interventional study: secondary analysis of RCT	Conventional versus high-intensity stepping training	Stroke < 6 months	36	Treadmill. Infrared. Motion Analysis. Modified Cleveland Clinic model	To evaluate the effects of up to 10 weeks of either high-intensity stepping training or conventional interventions on gait kinematics in individuals with subacute stroke.
Barroso, 2017 [59]	Observational study	/	Chronic stroke	9	Laboratory. Optoelectronic. Vicon. Plug In Gait model	To test the hypothesis that the combination of muscle synergies and biomechanical analysis will improve the functional assessment of walking performance post-stroke when compared to current clinical scales. The achievement of this goal could represent a key step towards a better quantitative assessment of walking post-stroke, and a deeper understanding of the cause-and-effect relationships between internal mechanisms and resulting functional performance.
Nikamp, 2017 [38] *	Interventional study: RCT	With/without AFO	Stroke < 6 weeks	33	Laboratory. Optoelectronic. Vicon. Modified Helen Hayes model	To study the effects of providing AFOs on two different moments in rehabilitation, early post-stroke.
Nikamp, 2018 [39] *	Interventional study: RCT	With/without AFO	Stroke < 6 weeks	26	Laboratory. Optoelectronic. Vicon. Modified Helen Hayes model	To study whether the patterns of recovery over time in terms of kinematics differed between early and delayed provision, and to study whether possible changes in kinematics or walking speed during the 26-week follow-up period differed between the groups.
Wang, 2018 [60] *	Interventional study: secondary analysis of RCT	Acupuncture	Stroke > 1–3 months	30	Laboratory. Infrared. Eagle 4, Motion Analysis. Custom model (pelvis and lower limbs)	To investigate the effect of acupuncture on changes in gait pattern associated with motor recovery in intracerebral haemorrhage patients.
Reissman, 2018 [43] *	Interventional study	Before/after treadmill rehabilitation with cross-tilt	Chronic stroke	12	Treadmill. Infrared. Motion Analysis. Custom model including trunk and head.	To explore whether a training paradigm that increases the demand for toe clearance during swing would enhance the ability to perform selective control between frontal and sagittal plane degrees-of-freedom, expressed as kinematic changes post-exposure.
Akbas, 2019 [61] **	Interventional study	Constrained stiff–knee gait in healthy subjects versus post-stroke subject gait analysis	Chronic stroke	9 from a previous study (Sulzer, 2010 [40])	Treadmill. Optical motion capture system (PhaseSpace Motion Capture, San Leandro, CA). Custom model (pelvis and lower limbs)	To simulate the kinematic constraints of those with SKG in unimpaired individuals and then compare against recorded data collected from participants with post-stroke SKG in a previous study.
Dean, 2020 [62]	Observational study	/	Chronic stroke	29	Dual-belt instrumented treadmill. 12-camera. Vicon. Custom full-body model	To investigate whether post-stroke changes in paretic propulsion magnitude or timing influence the swing phase kinematics of the paretic leg.
Silva, 2020 [32]	Observational study: retrospective	/	Unspecified	34	Laboratory. Optoelectronic. Vicon. Model not specified	To determine which gait parameters are associated with higher velocity in stroke patients with spastic paresis.
Van Criekinge, 2020 [63]	Observational study	/	Stroke < 6 months	57	Laboratory. Optoelectronic. Vicon. Plug In Gait model	To identify trunk abnormalities and differentiate between primary deviations and secondary compensations as far as possible, given that such differentiation is difficult to establish.
Daryabor, 2020 [19]	Observational study: case series	Walking with/without AFO	Chronic stroke	2	Laboratory. Optoelectronic. Vicon. Plug In Gait model	To design and evaluate a new articulated AFO incorporating a spring to determine its efficacy on spatiotemporal parameters, kinematics, and kinetics of lower-limb joints in two stroke patients.
Haruyama, 2021 [64]	Observational study	/	Chronic stroke	42	Laboratory. Optoelectronic. Vicon. Plug In Gait model	To provide a kinematic representative value by quantifying PTD and to clarify the PTD characteristics of hemiplegic gait compared to those of healthy subjects.
Sekiguchi, 2022 [65]	Observational study	On an even surface/on artificial grass	Post stroke	14	Laboratory, Optoelectronic, Mac 3D. Custom full-body model	To examine stepping patterns during gait on uneven surfaces in post-stroke patients and their relationship with real-world walking activity.
Nedergard, 2022 [30]	Observational study	/	Stroke > 3 months	31	Laboratory, Optoelectronic, Oqus. Custom full-body model	To contribute towards such a consensus by identifying a core set of a few kinematic variables to discriminate post-stroke gait from the gait of non-disabled controls.
Dumont-Casalechi, 2022 [45] *	Interventional study: RCT	Before/after PBMT–SMF	Chronic stroke > 6 months	10	Laboratory, Optoelectronic, SMART-D140. Plug In Gait model	To test different doses of PBMT–SMF, to identify the ideal dose to trigger immediate effects on the spatiotemporal and kinematic variables of gait in post-stroke individuals.
Dumont-Cimolin, 2022 [44] *	Interventional study: RCT	Before/after TDCs with treadmill training	Chronic stroke	14	Laboratory, Optoelectronic, SMART-D 140. Plug In Gait model	To investigate the effects of a single session and 10 sessions of anodal tDCS combined with treadmill training on spatiotemporal and kinematic gait variables in stroke survivors and determine whether these effects are maintained one month after the 10-session intervention has been completed.
Steffensen, 2023 [66]	Observational study: case series	/	Chronic stroke	3	Laboratory, Optoelectronic, Oqus. Custom model including trunk.	To quantify kinematic differences between marker-based and marker-less motion capture systems in individuals with impaired gait.
Kettlety, 2023 [67]	Observational study: secondary analysis of previous cross-sectional studies	/	Chronic post-stroke > 6 months	28	Treadmill. Optoelectronic. Vicon. Modified Cleveland Clinic model	To demonstrate the effect of fast walking on gait kinematics post-stroke relative to neurotypical adults, and to further define the advantages and limitations of this intervention in addressing gait biomechanics post-stroke.

AFO: ankle–foot orthosis; DAFO: dynamic ankle–foot orthosis; FES: functional electrical stimulation; MOCAP: motion capture system; PBMT-SMF: photobiomodulation therapy combined with static magnetic field. PTD: pelvic–toe distance; RCT: randomised controlled trial; SKG: stiff–knee gait; TBI: traumatic brain injury. * Studies included in the quantitative analysis. ** Studies not included in the quantitative analysis because of a lack of quantitative data available for analysis.

**Table 2:** List of relevant biomarkers.

Name of Biomarker	Definition of Biomarker
Hip_ROM_Sw	Hip ROM during swing
Hip_Max_Sw	Max hip flexion during swing phase
Hip_Toe-off	Hip flexion at toe-off
Hip_Max_TermSw	Max flexion at terminal swing
Hip_Max_Cycle	Max hip flexion during cycle
Knee_ROM_Sw	Knee flexion ROM during swing
Knee_Max_Sw	Max knee flexion during swing phase
Knee_Toe-off	Knee flexion at toe-off
Knee_Max_MidSw	Knee flexion at mid-swing
Knee_Max_Cycle	Max knee flexion during cycle
Ankle_ROM_Sw	Max ankle DF during swing
Ankle_Max_Sw	Ankle ROM during swing
Ankle_MidSw	Ankle angle at mid-swing
Min_PTD_Sw	Minimal pelvic–toe distance
Sag_Clear_Angle	Sagittal clearance angle
ToeClear_M5_MidSw	Vertical height of the marker placed on M5 relative to the foot flat on the ground at mid-swing
ToeClear_Max_Sw	Maximal toe clearance during swing (vertical displacement of the M2 toe marker)
ToeClear_Min_Sw	Minimal toe clearance during swing (vertical displacement of the M2 toe marker)
Front_Clear_Angle	Frontal clearance angle
HipAbd_ROM_Sw	Hip abduction ROM during swing
HipAbd_Max_Sw	Max hip abduction during swing
HipAbd_Max_Cycle	Max hip abduction during cycle
HipAbd_MidSw	Hip abduction at mid-swing
ThighAbd_Max_Sw	Max thigh abduction during swing
ThighAbd_Max_Cycle	Max thigh abduction during cycle
ThighAbd_MidSw	Thigh abduction at mid-swing
AnkleLateralDisp_Max_Sw	Max ankle lateral displacement during swing
AnkleLateralDisp_Max_Cycle	Max ankle lateral displacement during cycle
FootLateralDisp_Max_Sw	Max foot lateral displacement during swing
FootLateralDisp_Max_Cycle	Max foot lateral displacement during cycle
FootLateralDisp_MinToeClear	Foot lateral displacement at minimal toe clearance
PelvObl_ROM_Sw	Pelvic obliquity ROM during swing
PelvObl_Max_Sw	Max pelvic obliquity during swing
PelvObl_MidSw	Pelvic obliquity at mid-swing
HipHiking_Max_Sw	Hip hiking, defined as the vertical position of the ASIS marker calculated during quiet standing, was compared with the maximal vertical position during the swing phase
HipRot_ROM_Sw	Hip rotation ROM during swing
HipER_Max_Sw	Max hip external rotation during swing
PelvPostRot_Max_Sw	Maximal pelvic rotation during swing
PelvRot_MidSw	Pelvic backward rotation at mid-swing
Vault	Non-paretic max ankle plantar flexion during stance

ASIS: anterior superior iliac spine; M2: Second metatarsal; M5: fifth metatarsal; ROM: Range of motion.

**Table 3:** Classes of effect size of the limb-shortening and compensatory movement biomarkers based on the Hedges’ g effect size of therapeutic interventions (pre/post intervention and with/without AFO comparison). Data are shown as the number of studies (percentage of studies) [number of statistically significant Hedges’ g].

	Pre- and Post-Intervention Analyses							With and Without Orthosis Analysis						
Classes of size effects	*n*	<−0.8	−0.8 to –0.2	−0.2 to 0	0 to 0.2	0.2 to 0.8	>0.8	*n*	<−0.8	−0.8 to –0.2	−0.2 to 0	0 to 0.2	0.2 to 0.8	>0.8
Biomarkers of Limb Shortening														
Hip ToeOff	5	1 (20%) [1]	3 (60%) [1]	1 (20%)				6	1 (17%) [1]		3 (50%)	2 (33%)		
Hip Max Sw	8		3 (38%)	2 (25%)	1 (13%)	2 (25%)		8			1 (13%)	7 (88%)		
Hip Max Cycle	2		2 (100%)					0						
Hip ROM Sw	0							2				1 (50%)	1 (50%)	
Knee ToeOff	4			1 (25%)	2 (50%)	1 (25%)		6		1 (17%)	3 (50%)	2 (33%)		
Knee Max Sw	18			3 (17%)	8 (44%)	4 (22%)	3 (17%) [3]	9	1 (11%) [1]	2 (22%)	2 (22%)	4 (44%)		
Knee Max Cycle	3				1 (33%)	2 (67%)		0						
Ankle Max Sw	17		1 (6%)	2 (12%)	3 (18%)	9 (53%) [1]	2 (12%) [2]	10				1 (10%)	7 (70%) [4]	2 (20%) [2]
Ankle ROM Sw	0							2		2 (100%)				
ToeClear Min Sw	0							1			1 (100%)			
Biomarkers of Compensatory Movement														
PelvObl Max Sw	4		1 (25%)		3 (75%)			7		2 (29%)	5 (71%)			
PelvObl ROM Sw	0							2			2 (100%)			
HipHiking Max Sw	0							1		1 (100%)				
HipAbd Max Sw	4	1 (25%) [1]	3 (75%)					5			2 (40%)	2 (40%)	1 (20%)	
HipAbd Mid Sw	1					1 (100%)		0						
HipAbd ROM Sw	0							2		2 (100%)				
HipAbd Max Cycle	15		1 (7%) [1]	2 (13%)	4 (27%)	6 (40%)	2 (13%) [2]	0						
FootLateralDisp Max Sw	0							1		1 (100%)				
FootLateralDisp Max Cycle	1	1 (100%) [1]						0						
AnkleLateralDisp Max Cycle	3					2 (67%)	1 (33%) [1]	0						
HipER Max Sw	1			1 (100%)				0						

**Table 4:** Recommendation of kinematic biomarkers to describe limb shortening and compensatory movements in hemiparetic gait. The italic font refers to hip biomarkers and indicates that the thigh biomarker should be used.

	Joints and Segments	Shortening Biomarkers	Compensatory Biomarkers	Standard Name of Compensation
Sagittal plane	*Hip*	*Maximal flexion during swing*	*Maximal flexion during swing*	Stepping
	Thigh	Maximal elevation angle during swing	Maximal elevation angle during swing	
	Knee	Maximal flexion during swing	Maximal flexion during swing	
	Ankle	Maximal dorsiflexion during swing	Contralateral angle at mid-stance	Vaulting
	Limb length	Minimal hip–toe distance during swing normalised by bilateral stance (%)		
Frontal plane	Pelvis		Maximal pelvic upward obliquity during swing	Hip hiking
			Maximal elevation of the hip during swing compared to its position during the previous stance (cm)	Hip hiking + vaulting
	*Hip*		*Maximal hip abduction during swing*	Circumduction
	Thigh		Maximal thigh abduction during swing	
	Ankle		Maximal ankle lateral displacement during swing (cm)	
Transversal plane	Hip		Maximal lateral rotation during swing	
	Pelvis		Maximal posterior rotation during swing	Pelvic posterior rotation

## Data Availability

Data extraction tables can be provided by the authors upon reasonable request.

## Supplementary Materials

The following supporting information can be downloaded at: https://www.mdpi.com/article/10.3390/s25154598/s1. Table S1: Lower limb biomarkers in the sagittal plane deemed irrelevant for describing limb shortening during the swing phase. Table S2: Lower limb and pelvis biomarkers in the frontal and transversal planes, and pelvis biomarkers in the sagittal plane deemed irrelevant for describing compensatory movements. Table S3: Characteristics of studies excluded from qualitative analysis. Table S4: Bias assessed according to JBI’s critical appraisal tools. Table S5: Quantitative analysis based on the Hedges’ g effect size of therapeutic interventions (pre- and post-intervention and with/without AFO comparison).

## Abbreviations

The following abbreviations are used in this manuscript:

3D-IGA	Three-dimensional instrumental gait analysis
AFO	Ankle–foot orthosis
ASIS	Anterior superior iliac spine
DAFO	Dynamic ankle–foot orthosis
FES	Functional electrical stimulation
M2	Second metatarsal
M5	Fifth metatarsal
MOCAP	Motion capture system
PBMT-SMF	Photobiomodulation therapy combined with static magnetic field.
PTD	Pelvic–toe distance
RCT	Randomised controlled trial
ROM	Range of motion
SKG	Stiff–knee gait
TBI	Traumatic brain injury

## Appendix A

### Research Strategy

Pubmed: (hemipleg*[tw] OR hemipare*[tw] OR hemiparaly*[tw] OR stroke[tw] OR “Cerebrovascular Accident”[tw] OR CVA[tw] OR “brain vascular disease”[tw] OR “brain injury”[tw]) AND (Gait[tw]) AND (“three-dimensional”[tw] OR “quantitative gait analysis”[tw] OR kinematics[tw] OR kinematic[tw])

Cochrane, Scopus, PEDro, and Web of Science: (hemipleg* OR hemipare* OR hemiparaly* OR stroke OR “cerebrovascular accident” OR CVA OR “brain vascular disease” OR “brain injury”) AND gait AND (“three-dimensional” or “quantitative gait analysis” OR kinematics OR kinematic)
