# Supplementary material for: Kinematic Biomarkers of Limb Shortening and Compensations in Hemiparetic Gait: A Systematic Review
Source: Sensors (Basel). 2025 Jul 25;25(15):4598. doi: 10.3390/s25154598 (PMC12349382; doi:10.3390/s25154598)
Supplement: Supplementary file 1 [file sensors-25-04598-s001.zip › sensors-3731955-supplementary.pdf]

sensors

Table S1 - *Lower limb biomarkers in the sagittal plane deemed irrelevant for describing limb shortening during the swing phase.*

Hip	Knee	Ankle	Justification
Hip ROM during GC<br>Hip mean during cycle<br>Thigh elevation ROM during GC<br>Difference in hip F/E ROM between paretic and NP side<br>Hip ROM symmetry index during GC	Knee ROM during GC<br>Knee mean during cycle<br>Shank elevation ROM during GC<br>Difference in knee F/E ROM between paretic and NP side<br>Knee ROM symmetry index during GC	Ankle ROM during GC<br>Ankle mean during cycle<br>Foot elevation ROM during GC<br>Ankle ROM symmetry index during GC<br><br>**Other**<br>Limb length symmetry index during cycle	Not specific of swing phase (n = 15 biomarkers)
Hip angle at IC<br>Hip max extension during stance<br>Hip ROM during stance<br>Hip max flexion during loading response<br>Hip max extension during unipodal stance<br>Hip ROM during unipodal stance<br>Hip max flexion during stance<br>Hip ROM from IC to peak hip ext<br>Hip angle at 0-20% of GC<br>Hip angle at IC asymmetry ratio<br>Hip max extension during final stance	Knee angle at IC<br>Knee angle at pre-swing<br>Knee max extension during stance<br>Knee ROM during stance<br>Knee max flexion during loading response<br>Knee minimal flexion in terminal stance<br>Knee max extension during unipodal stance<br>Knee mean during stance<br>Knee ROM from peak knee flex in load response to peak knee ext in stance<br>Knee angle at IC asymmetry ratio<br>Knee max flexion during stance asymmetry ratio<br>Knee min angle during mid stance	Ankle angle at IC<br>Ankle max PF during stance<br>Ankle max DF during stance<br>Ankle ROM during stance<br>Ankle max PF during loading response<br>Ankle max DF during unipodal stance<br>Ankle max PF during unipodal stance<br>Ankle ROM during unipodal stance<br>Ankle ROM during loading response<br>Ankle ROM from peak DF during stance to peak PF during initial Sw<br>Ankle angle at 0-20% of GC<br>Ankle angle at IC asymmetry ratio<br>Ankle max DF during stance asymmetry ratio<br>Ankle max PF during pre-swing	Wrong time of gait cycle (n = 37 biomarkers)
NP Thigh elevation ROM during GC<br>NP Hip mean during cycle<br>Hip min flexion during swing<br>Hip max extension during GC<br>NP Hip F/E ROM<br>NP Hip max flexion during GC<br>NP Hip min flexion during GC<br>NP Hip angle at toe-off<br>NP Hip F/E profile<br>Thigh min angle during GC<br>NP Thigh min angle during GC<br>Hip max extension during GC asymmetry ratio<br>Hip max flexion during swing asymmetry ratio<br><br>**Other**<br>GH F/E ROM during GC<br>GH max flexion during GC<br>GH min flexion during GC<br>GH mean angle during GC<br>NP GH F/E ROM during GC<br>NP GH max flexion during GC<br>NP GH min flexion during GC<br>NP GH mean angle during GC	NP Shank elevation ROM during GC<br>Knee angle at highest position of centre of mass during NP stance<br>NP Knee mean during cycle<br>Knee min flexion during swing<br>Knee max extension during GC<br>NP Knee max flexion during swing<br>NP Knee F/E ROM<br>NP Knee max flexion during GC<br>NP Knee min flexion during GC<br>NP Knee angle at toe-off<br>NP Knee F/E profile<br>Knee max extension during GC asymmetry ratio<br>Knee max flexion during swing asymmetry ratio<br><br>**Other**<br>Elbow F/E ROM during GC<br>Elbow max flexion during GC<br>Elbow min flexion during GC<br>Elbow mean angle during GC<br>NP Elbow F/E ROM during GC<br>NP Elbow max flexion during GC<br>NP Elbow min flexion during GC	Ankle Max DF during GC<br>NP Foot elevation ROM during GC<br>Ankle angle at highest position of centre of mass during NP stance<br>Ankle angle at toe-off<br>NP Ankle mean during cycle<br>Ankle min DF during swing<br>Ankle max PF during GC<br>NP Ankle max DF during swing<br>NP Ankle DF/PF ROM<br>Ankle max PF during swing<br>NP Ankle max DF during GC<br>NP Ankle max PF during GC<br>NP Ankle angle at toe-off<br>NP Ankle DF/PF profile<br>Total between ankle flexion at IC and ankle flexion at toe-off<br>Foot min angle during GC<br>NP Foot min angle during GC<br>Foot max angle during GC<br>Ankle max DF during swing asymmetry ratio<br><br>**Other**<br>Trailing limb angle	Not limb shortening (n = 66 biomarkers)

	NP Elbow mean angle during GC	NP Trailing limb angle<br>Leg min angle during GC<br>NP Leg min angle during GC<br>Leg extension angle symmetry index	
Knee F/E profile<br>Hip F/E profile every 2%<br>Thigh F/E profile	Knee F/E profile<br>Knee F/E profile every 2%<br>Shank F/E profile	Ankle DF/PF profile<br>Ankle DF/PF profile every 2%<br>Foot F/E profile	Indiscrete analysis<br>(n = 9 biomarkers)

NP: non paretic, GH: glenohumeral, DF: dorsiflexion, PF: plantarflexion, F: flexion, E: extension, GC: gait cycle, ROM: range of motion, IC: initial contact, Sw: swing, Min: minimal, Max: maximal

Table S2. *Lower limb and pelvis biomarkers in the frontal and transversal planes, and pelvis biomarkers in the sagittal plane deemed irrelevant for describing compensatory movements (circumduction, hip hiking, pelvis posterior tilt, pelvic posterior rotation and vault).*

Frontal plane	Transversal plane	Sagittal plane	Justification
Pelvic obliquity ROM during GC<br>Pelvic obliquity max during GC<br>Pelvic obliquity min during GC<br>Hip Abd/Add ROM during GC<br>Hip Abd/Add mean during GC<br>Hip Abd min during GC<br>Hip Abd/Add ROM during GC symmetry index<br>Knee Abd/Add ROM during GC<br>Knee Abd/Add mean during GC<br>Knee Abd min during GC<br>Knee Abd max during GC<br>Ankle Abd/Add ROM during GC<br>Ankle Abd/Add mean during GC<br>Ankle Abd min during GC<br>Ankle Abd Max during GC	Pelvic anterior rotation max during GC<br>Pelvic posterior rotation max during GC<br>Pelvic ant/post rotation ROM during GC<br>Pelvic ant/post rotation mean during GC<br>Pelvic ant/post rotation at maximum CoM position with NP limb in stance<br>Hip ER max during GC<br>Hip ER min during GC<br>Hip ER/IR ROM during GC<br>Hip ER/IR mean during GC<br>Knee ER max during GC<br>Knee ER min during GC<br>Knee ER/IR ROM during GC<br>Knee ER/IR mean during GC<br>Ankle ER max during GC<br>Ankle ER min during GC<br>Ankle ER/IR ROM during GC<br>Ankle ER/IR mean during GC<br>Foot progression mean during GC<br>Foot progression ROM during GC	Pelvic posterior tilt max during GC<br>Pelvic anterior tilt max during GC<br>Pelvic ant/post tilt ROM during GC<br>Pelvic ant/post tilt mean during GC<br>Pelvic ant/post tilt at maximum CoM position with NP limb in stance<br>Pelvic anterior tilt max during GC asymmetry ratio	Not specific of swing phase (n = 40 biomarkers)
Pelvic obliquity at IC<br>Pelvic obliquity at toe-off<br>Pelvic obliquity at pre-swing<br>Pelvic obliquity min during stance<br>Pelvic obliquity max during stance<br>Pelvic obliquity ROM during stance<br>Hip Abd/Add ROM during stance<br>Hip Abd/Add at IC<br>Hip Abd/Add at toe-off<br>Hip Abd/Add at pre-swing<br>Hip Add max during stance<br>Hip Abd max during stance<br>Hip Add max during mid-stance<br>Knee Abd max during stance<br>Knee Abd min during stance<br>Ankle Abd max during stance<br>Upper body inclination angle during stance<br>Lower body inclination angle during stance	Pelvic rotation at IC<br>Pelvic rotation ROM during stance<br>Hip ER/IR ROM during stance<br>Hip rotation at IC<br>Hip IR max during stance<br>Hip IR max during mid-stance<br>Hip ER/IR mean during stance<br>Ankle rotation at IC<br>Ankle rotation at toe-off<br>Ankle ER max during mid-stance<br>Ankle ER max during stance<br>Foot progression at IC<br>Foot progression at toe-off<br>Foot progression mean during stance	Pelvic tilt at IC<br>Pelvic tilt at foot-off<br>Pelvic tilt at NP IC<br>Pelvic tilt at NP foot-off	Wrong time of gait cycle (n = 36 biomarkers)
Hip Add max during swing<br>NP Hip Abd max during stance<br>NP Hip Abd max during GC<br>NP Hip Abd min during GC<br>NP Hip Abd/Add ROM during GC<br>NP Hip Abd/Add mean during GC<br>NP Knee Abd max during GC<br>NP Knee Abd min during GC<br>NP Knee Abd/Add ROM during GC<br>NP Knee Abd/Add mean during GC<br>NP Ankle Abd max during GC	Pelvic ER min during mid-swing<br>Pelvic ER/IR ROM during stance of NP limb<br>NP Hip ER max during GC<br>NP Hip ER min during GC<br>NP Hip ER/IR ROM during GC<br>NP Hip ER/IR mean during GC<br>NP Knee ER max during GC<br>NP Knee ER min during GC<br>NP Knee ER/IR ROM during GC<br>NP Knee ER/IR mean during GC	Pelvic anterior tilt max during swing<br>Elbow F/E max during GC<br>Shoulder F/E max during GC<br>Elbow F/E ROM during GC<br>Shoulder F/E ROM during GC<br>Thorax F/E ROM during GC<br>Pelvic ant/post tilt ROM during GC<br>Elbow F/E ROM index during GC<br>Shoulder F/E ROM index during GC<br>Thorax F/E ROM index during GC	Wrong movement analysis (n = 104 biomarkers)

NP Ankle Abd min during GC<br>NP Ankle Abd/Add ROM during GC<br>NP Ankle Abd/Add mean during GC<br>Ankle Add max during swing<br>GH Abd max during GC<br>GH Abd min during GC<br>GH Abd/Add ROM during GC<br>GH Abd/Add mean during GC<br>NP GH Abd max during GC<br>NP GH Abd min during GC<br>NP GH Abd/Add ROM during GC<br>NP GH Abd/Add mean during GC<br>Elbow Abd max during GC<br>Elbow Abd min during GC<br>Elbow Abd/Add ROM during GC<br>Elbow Abd/Add mean during GC<br>NP Elbow Abd max during GC<br>NP Elbow Abd min during GC<br>NP Elbow Abd/Add ROM during GC<br>NP Elbow Abd/Add mean during GC<br>NP Foot lateral displacement during GC<br>Hip Abd/Add ROM index during GC<br>Shoulder Abd/Add ROM during GC<br>Thorax Abd/Add ROM during GC<br>Shoulder Abd/Add ROM index during GC<br>Thorax Abd/Add ROM index during GC<br>Shoulder Abd max during GC<br>Lower body inclination angle during Sw<br>Upper body inclination angle during Sw<br>Thorax Abd max during GC<br>Thorax Abd min during GC<br>Lateral displacement of the pelvis ROM during GC<br>Lateral displacement of the CoM ROM during GC	NP Ankle ER max during GC<br>NP Ankle ER min during GC<br>NP Ankle ER/IR ROM during GC<br>NP Ankle ER/IR mean during GC<br>Ankle IR max during swing<br>Ankle ER max during swing<br>GH ER max during GC<br>GH ER min during GC<br>GH ER/IR ROM during GC<br>GH ER/IR mean during GC<br>NP GH ER max during GC<br>NP GH ER min during GC<br>NP GH ER/IR ROM during GC<br>NP GH ER/IR mean during GC<br>Elbow ER max during GC<br>Elbow ER min during GC<br>Elbow ER/IR ROM during GC<br>Elbow ER/IR mean during GC<br>NP Elbow ER max during GC<br>NP Elbow ER min during GC<br>NP Elbow ER/IR ROM during GC<br>NP Elbow ER/IR mean during GC<br>Hip ER/IR ROM index during GC<br>Shoulder ER/IR ROM during GC<br>Thorax ER/IR ROM during GC<br>Shoulder ER/IR ROM index during GC<br>Thorax ER/IR ROM index during GC<br>Shoulder ER max during GC<br>Thorax ER max during GC<br>Thorax ER min during GC<br>Foot progression min during GC	Pelvic ant/post tilt ROM index during GC<br>Thorax flexion max during GC<br>Thorax flexion min during GC<br>Thorax flexion max during swing<br>Thorax flexion at IC<br>Thorax flexion at NP foot off<br>Thorax flexion at NP IC<br>Thorax flexion at foot off<br>Hip flexion max during swing as compensation of drop foot (steppage)	
Toe mediolateral displacement profile during GC<br>Thorax Abd/Add profile<br>Shoulder Abd/Add Profile<br>Pelvic obliquity profile<br>Hip Abd/Add profile<br>Knee Abd/Add profile<br>Ankle Abd/Add profile<br>NP Pelvic obliquity profile<br>NP Hip Abd/Add profile<br>NP Knee Abd/Add profile<br>NP Ankle Abd/Add profile<br>Pelvic obliquity profile every 2%<br>Hip Abd/Add profile every 2%	Thorax ER/IR profile<br>Pelvic ER/IR profile<br>Hip ER/IR profile<br>Knee ER/IR profile<br>Ankle ER/IR profile<br>Foot progression ER/IR profile<br>NP Pelvic ER/IR profile<br>NP Hip ER/IR profile<br>NP Knee ER/IR profile<br>NP Ankle ER/IR profile<br>Pelvic ER/IR profile every 2%<br>Hip ER/IR profile every 2%<br>Foot progression ER/IR profile every 2%	NP Shoulder F/E profile<br>NP Elbow F/E profile<br>Pelvic ant/post tilt profile<br>NP Pelvic ant/post tilt profile<br>Pelvic ant/post tilt profile every 2%	Indiscrete analysis (n = 31 biomarkers)

NP: non paretic, GH: glenohumeral, DF: dorsiflexion, PF: plantarflexion, F: flexion, E: extension, Abd: abduction, Add: adduction, ER: external rotation, IR: internal rotation, Ant: anterior, Post: posterior, GC: gait cycle, ROM: range of motion, IC: initial contact, Sw: swing, CoM: centre of mass, Min: minimal, Max: maximal

Table S3 *- Characteristics of studies excluded from qualitative analysis following biomarkers selection*

Author	Design	Intervention	System analysis	Main objective
**Studies with limb-shortening description only (n=15)**				
Yamamoto, 2018	Interventional study: RCT	With/without AFO	Laboratory. Vicon.	To investigate the effect of the plantar flexion resistance of AFOs on the gait of stroke patients in the subacute phase.
Chen, 2003	Observational study	/	Laboratory. Optoelectronic. Vicon	To identify the compensatory adaptations of pelvic motion within and between limbs in stroke patients with different degrees of motor recovery.
Little, 2018	Observational study	/	Treadmill. Infrared. Vicon	To develop a classification system for hemiparetic gait dysfunction based on the magnitude of pelvic excursion deviation.
Hwang, 2017	Interventional study	4 conditions: no sling/a flexed sling/an extended arm sling/and an elastic arm sling	Laboratory. Optoelectronic. Vicon	To investigate how the muscle activities of the affected arm and kinematic data taken during walking are influenced by flexion-type (hemisling), extension-type (Rolyan sling), and elastic arm slings under elastic tension.
De Luca, 2013	Interventional study	Before/after robot end-point assisted gait re-education	Laboratory. Infrared. BTS Smart System.	To investigate the effects of robot training in the different planes - sagittal, frontal, transverse - and the interaction between the motion of the “affected” and “unaffected” limb.
Aneiros-Tarancon, 2017	Observational study: case report	Walk with/without cognitive task	Laboratory. Optoelectronic. Vicon	To analyse the changes occurring in the kinematic gait pattern of a 57-year-old man with stroke in response to a concurrent cognitive task.
Michalina, 2017	Observational study	/	Laboratory. Optoelectronic. Vicon	To identify compensatory mechanisms in patients with drop foot.
Caty, 2009	Observational study	/	Treadmill. Infrared. Elite.	To assess the reliability of kinematic, mechanical and energetic gait variables at short (1 day) and medium (1 month) intervals in adult patients after stroke.
Straudi, 2009	Observational study: validation of classification	/	Laboratory. Optoelectronic. Vicon	To classify a sample of chronic hemiplegic subjects in their level of walking performance; to identify different kinematic profiles and ambulation classes in the sub-groups defined.
Yavuzer, 2007	Interventional study: RCT	Before/after conventional rehabilitation + 20 sessions of SES versus conventional rehabilitation + 20 sessions of sham SES	Laboratory. Optoelectronic. Vicon	To evaluate the effects of sensory-amplitude electric stimulation (SES) of the paretic leg on motor recovery and gait kinematics of patients with stroke.
Yavuzer, 2008	Observational study: metrology	/	Laboratory. Optoelectronic. Vicon	To assess the repeatability of the time–distance parameters and sagittal plane gait kinematics of patients with stroke.
Yavuzer, Geler, 2006	Interventional study: RCT	Before/after conventional rehabilitation program versus conventional rehabilitation + NMES	Laboratory. Optoelectronic. Vicon	To evaluate the effects of neuromuscular electric stimulation (NMES) of the tibialis anterior muscle on motor recovery and gait kinematics of patients with stroke.
Yao, 2017	Observational study: retrospective	Before/after implantation of foot drop stimulator	Laboratory. Optoelectronic. Vicon.	To examine with a larger cohort whether the use of iPNS induces a lower-extremity flexion withdrawal response evidenced by increased knee and hip flexion during the swing phase or whether the use of iPNS reduces compensatory movements by decreasing knee flexion and hip circumduction as these are no longer necessary to equal the relative leg extension due to drop foot.

Jurkojc, 2012	Observational study	/	Laboratory. Camcorder. Apas System	The article focuses on research into gait kinematics of individuals post-stroke. Comparative analyses of angle courses and gait parameters were carried out. Results obtained for individuals with left- and right-sided paresis were compared.
Daryabor, 2021	Interventional study: RCT	2 types of AFO	Laboratory. Optoelectronic. Vicon.	To evaluate the effect of two ankle-foot orthoses (AFOs), AFO with plantar flexion stop (AFO-PlfS) and AFO with plantar flexion resistance (AFO-PlfR), while wearing standard shoes and rocker-sole shoes.
Daryabor 2022	Interventional study	With/without AFO	Laboratory, Optoelectronic, Oqus	To contribute toward such a consensus by identifying a core set of a few kinematic variables to discriminate post-stroke gait from the gait of non-disabled controls.
**Studies with compensatory movement description only (n=1)**				
Wang, Hou, 2020	Interventional study: RCT	Acupuncture	Laboratory. 3D gait analysis (Eagle 4)	To evaluate the additional effects of acupuncture treatment on motor function in patients with subacute haemorrhagic stroke.
**Studies with no limb shortening or compensation description (n=25)**				
Wallard, 2018	Observational study	/	Treadmill. Infrared. Elite.	To evaluate how severe hip osteoarthritis affects lower-limb coordination during gait by using the KSC law method and predict the energy expenditure.
Barton, 2012	Observational study: validation of gait index	/	/	To determine how the Gillette Gait Index could be used to assess gait abnormalities in adults with central nervous system disorders.
De Luca, 2019	Interventional study: retrospective	Gait training with and without endpoint robot	Laboratory. Infrared cameras. DAVIS protocol. SMART.	To understand the extent to which a potential post-training improvement in gait speed is due to the real recovery of lost walking abilities or it is related to changes in compensatory strategies?
Kim, 2004	Observational study	/	Laboratory. Optoelectronic. IRED system.	To establish some types of kinematic and kinetic gait patterns (based on shape and direction of curves) in the frontal, transverse and sagittal profiles exhibited by individuals with chronic stroke. Secondly, does the magnitude or pattern of these profiles relate to gait speed?
Lamontagne, 2004	Interventional study	4 walking conditions: full weight-bearing at preferred and maximal speed/body weight support at preferred and maximal speed	Laboratory. Optoelectronic. Vicon	To investigate the extent to which stroke subjects can increase their speed and modify their gait pattern during (1) fast walking, (2) BWS and (3) combined fast walking and BWS.
Guzik, 2018	Observational study	/	Laboratory. Infrared. BTS Smart System.	To investigate the correlations between spatiotemporal parameters acquired in 3DGA and spatiotemporal traits assessed as subscale 1 of the WGS. To compare the 3D kinematic parameters and the kinematic parameters evaluated by subscales 1, 2, 3 and 4 of the WGS.
Hwang, 2010	Interventional study	Before/after locomotor imagery training versus conventional rehabilitation	Laboratory. Optoelectronic. Vicon	To evaluate whether motor imagery for gait training improves spatiotemporal parameters on gait, related kinematic gait variables and clinical measures for gait in individuals with hemiparetic stroke.
Mah, 1999	Observational study	/	Analysis.	To determine whether our quantitative kinematic gait pattern analysis would characterise functional recovery after neurological injury more completely

			Laboratory. Optoelectronic. ELITE	than easily measurable quantities such as gait speed and joint range of motion.
Yavuzer, 2006	Interventional study: RCT	Before/after conventional rehabilitation versus conventional rehabilitation + balance training	Laboratory. Optoelectronic. Vicon	To investigate the effects of balance training using force platform biofeedback on quantitative gait characteristics of hemiparetic patients late post-stroke.
Kuan, 1999	Observational study	/	Laboratory. Infrared. SUN PARC system.	To study the effect of cane use on the gait of hemiplegic patients.
Kim, 2015	Observational study	/	Laboratory. Optoelectronic. Vicon	To compare the gait of chronic ambulatory hemiplegic elderly (HE) subjects with that of non-hemiplegic elderly (NHE) subjects using the Vicon® 512 motion analysis system to track movement trajectories.
Williams, Lai, 2015	Observational study: Validation of classification	/	Laboratory. Optoelectronic. Vicon	To classify gait disorders following TBI on the basis of pelvic and lower limb kinematic data. Therefore, the aim of this study was to develop a classification system for TBI gait disorders and to determine its accuracy.
Massaad, 2010	Interventional study: pilot	Before/after training with vertical CM displacement biofeedback	Treadmill. Optoelectronic. BTS Smart System.	We tested a new rehabilitation strategy to reduce walking energy costs in hemiparetic patients by helping them to actively reduce excessive vertical CM (centre of mass) displacement through biofeedback.
Oken, 2008	Observational study: retrospective	/	Laboratory. Optoelectronic. Vicon	To determine the asymmetry ratio of spatio-temporal and kinematic gait data in patients with stroke, and to compare the subgroups (based on gender, time since stroke, lesion type, side of paresis, motor recovery, sensory status and walking velocity) in terms of the asymmetry ratio.
Caillet, 2003	Observational study	/	Laboratory. Optoelectronic. Vicon	To assess the interest of three-dimensional gait analysis in measuring the effect of the orthosis and of selective tibial neurotomy without visual analysis.
Sousa, 2009	Interventional study	3 walking conditions: no harness/with harness and 0% weight-bearing/with harness and 30% body weight support	Laboratory. Digital cameras. APAS system	To investigate individuals with chronic stroke, walking overground with BWS.
Shin, 2021	Observational study	/	Laboratory, Optoelectronic, Vicon	To investigate the relationship between modular neuromuscular deficits and disturbances in gait quality measures (i.e., asymmetry) in terms of spatiotemporal, limb and joint kinematic parameters in chronic post-stroke individuals.
Kim, 2016	Observational study	/	Laboratory. Optoelectronic. Vicon	To identify the kinematic differences of the primary and secondary joints of stroke survivors.
Schwartz, 2008	Observational study: validation of a gait index	/	Laboratory, Optoelectronic. Motion Analysis.	This article describes a new multivariate measure of overall gait pathology referred to as the Gait Deviation Index (GDI).
Kaczmarczyk 2009	Observational study: validation of classification	/	Laboratory. Analogue cameras. APAS system	To test three methods for classifying post-stroke patients into gait pattern types, based around the three types of foot position presented by Wong *et al*. The following methods were considered: (1) qualitative test results of gait kinematics as well as two different types of quantitative investigation: (2) min/max joint angle values and (3) the full progression of joint angle changes.

Prado-Medeiros, 2011	Interventional study	Before/after BWS training with and without FES	Laboratory. Analogue cameras. APAS system	To investigate the effects of adding FES to the common peroneal nerve on ground level gait training with BWS, on spatio-temporal gait parameters, segmental angles and motor function in chronic stroke patients.
Cho, 2022	Interventional study: RCT	Before/after training with electrical stimulation versus conventional rehabilitation	Laboratory. Optoelectronic. VICON.	To investigate the effects of passive biaxial ankle movement training (AMT) synchronised with EST (AMT-EST) on ankle proprioception, pROM, strength and functional performance, including lower extremity impairment, balance and gait.
Mao, 2022	Interventional study: RCT	Before/after training with transcutaneous peroneal nerve stimulator versus conventional home and/or community gait training therapy	Laboratory. Optoelectronic. VICON.	To explore the efficacy and safety of tPNS for facilitating physiological ankle and foot movement in a therapeutic setting using changes in the participants' mechanistic outcomes and kinematic and kinetic parameters.
Daryabor, 2022	Interventional study	Before/after rehabilitation wearing AFO-PS or AFO-OD	Laboratory. Optoelectronic. C-Motion.	To demonstrate the therapeutic effect of gait training using ankle-foot orthoses (AFOs) on the gait of stroke patients when not wearing AFOs with two different types of AFO, an AFO with an oil damper (AFO-OD) that resists plantarflexion and an AFO with a plantarflexion stop (AFO-PS), and to display the possible differences between AFO types.
Van Bladdel, 2022	Observational study	/	Laboratory. Optoelectronic. VICON.	To compare spatiotemporal (including variability and symmetry measures), kinematic and kinetic gait parameters between self-paced and fixed-speed treadmill walking in individuals post-stroke.

Table S4. *Bias assessed according to JBI's critical appraisal tools*

**Quasi experimental studies**	Is it clear in the study what is the 'cause' and what is the 'effect' (i.e. there is no confusion about which variable comes first)?	Were the participants included in any similar comparisons?	Were the participants included in any comparisons receiving similar treatment/care other than the exposure or intervention of interest?	Was there a control group?	Were there multiple measurements of the outcome both pre- and post- intervention/exposure?	Was follow-up complete and if not, were differences in inter-group follow-up adequately described and analysed?	Were the outcomes of participants included in any comparisons measured in the same way?	Were outcomes measured in a reliable way?	Was statistical analysis used appropriately?
Cruz, 2009	Yes	Yes	Yes	No	Yes	Yes	Yes	Yes	Yes
Boudarham, 2014	Yes	Yes	Yes	No	Yes	Yes	Yes	Yes	Yes
Qian, 2015	Yes	Yes	Yes	No	Yes	Yes	Yes	Yes	Yes
Shin, 2015	Yes	Yes	Yes	No	Yes	Yes	Yes	Yes	Yes
Awad, 2017	Yes	Yes	Yes	No	Yes	Yes	Yes	Yes	Yes

**RCT studies**	Was true randomisation used to assign participants to treatment groups?	Was allocation to treatment groups concealed?	Were treatment groups similar at baseline?	Were participants blind to treatment assignment?	Were those delivering the treatment blind to treatment assignment?	Were treatment groups treated identically other than with regard to the intervention of interest?	Were outcome assessors blind to treatment assignment?	Were outcomes measured in the same way for treatment groups?	Were outcomes measured in a reliable way?	Was follow up complete and if not, were differences in inter-group follow-up adequately described and analysed?	Were participants analysed in the groups to which they were randomised?	Was statistical analysis used appropriately?	Was the trial design appropriate and were any deviations from the standard RCT design (individual randomisation, parallel groups) accounted for in the conduct and analysis of the trial?
Mahtani, 2017	Yes	No	Yes	No	No	Yes	No	Yes	Yes	Yes	Yes	Yes	Yes
Nikamp, 2017	Yes	No	Yes	No	No	Yes	No	Yes	Yes	Yes	Yes	Yes	Yes
Nikamp, 2018	Yes	No	Yes	No	No	Yes	No	Yes	Yes	Yes	Yes	Yes	Yes
Wang, 2018	Yes	Yes	Yes	Yes	No	Yes	Yes	Yes	Yes	Yes	Yes	Yes	Yes
Dumont-Cimolin, 2022	Yes	Yes	Yes	Yes	Yes	Yes	Yes	Yes	Yes	Yes	Yes	Yes	Yes
Dumont-Casalechi, 2022	Yes	Yes	Yes	Yes	Yes	Yes	Yes	Yes	Yes	Unclear	Yes	Yes	Yes

**Case reports**	Were patient demographic characteristics clearly described?	Was the patient's history clearly described and presented as a timeline?	Was the current clinical condition of the patient on presentation clearly described?	Were diagnostic tests or assessment methods and results clearly described?	Was/were the intervention(s) or treatment procedure(s) clearly described?	Was the post-intervention clinical condition clearly described?	Were adverse events (harms) or unanticipated events identified and	Does the case report provide takeaway lessons?		
Nolan, 2010	Yes	Yes	Yes	Yes	Yes	Yes	Yes	Yes		
**Case series**	Were there clear criteria for inclusion in the case series?	Was the condition measured in a standard, reliable way for all participants included in the case series?	Were valid methods used to identify the condition of all participants included in the case series?	Did the case series have consecutive inclusion of participants?	Did the case series have complete inclusion of participants?	Were the demographics of the study participants clearly reported?	Was the participants' clinical information clearly reported?	Were the outcomes or follow-up results of cases clearly reported?	Was the demographic information of the presenting site(s)/clinic(s) clearly reported?	Was the statistical analysis appropriate?
Daryabor, 2020	Yes	Yes	Yes	No	Yes	Yes	Yes	Yes	Yes	No
**Analytical cross-sectional studies**	Were the criteria for inclusion in the sample clearly defined?	Were the study subjects and the setting described in detail?	Was the exposure measured in a valid and reliable way?	Were objective, standard criteria used to measure the condition?	Were confounding factors identified?	Were strategies to deal with confounding factors outlined?	Were the outcomes measured in a valid and reliable way?	Was statistical analysis used appropriately?		
Zollo, 2015	Yes	Yes	Yes	Yes	No	No	Yes	Yes		

Table S5. *Quantitative analysis based on the Hedges' g effect size of therapeutic interventions (pre- and post-intervention and with/without AFO comparison)*

**Study**	**Subgroup**	**Shortening biomarkers**	**Hedges' g (lower to upper 95% CI)**	**Compensation biomarkers**	**Hedges' g (lower to upper 95% CI)**	**Biomarkers most sensitive to change (significant size effect)**
**Pre and post intervention comparison**						
Nikamp, 2018	Early AFO / No AFO	Hip Max Sw<br>Knee Max Sw<br>Ankle Max Sw<br>Hip Toe-Off<br>Knee Toe-Off	-0.36(-0.87 to 0.15)<br>0.14(-0.36 to 0.63)<br>0.36(-0.15 to 0.87)<br>-0.72(-1.28 to -0.16)*<br>0.12(-0.38 to 0.61)	Pelv Obl Max Sw<br>Hip Abd Max Sw	0.16(-0.33 to 0.66)<br>-0.30(-0.81 to 0.20)	Hip Toe-Off
Nikamp, 2018	Delayed AFO / No AFO	Hip Max Sw<br>Knee Max Sw<br>Ankle Max Sw<br>Hip Toe-Off<br>Knee Toe-Off	-0.28(-0.94 to 0.38)<br>0.37(-0.30 to 1.04)<br>-0.19(-0.84 to 0.46)<br>-0.46(-1.14 to 0.23)<br>0.25(-0.41 to 0.91)	Pelv Obl Max Sw<br>Hip Abd Max Sw	0.03(-0.61 to 0.67)<br>-0.87(-1.66 to -0.08)*	HipAbd Max Sw
Nikamp, 2018	Early AFO / With AFO	Hip Max Sw<br>Knee Max Sw<br>Ankle Max Sw<br>Hip Toe-Off<br>Knee Toe-Off	-0.29(-0.80 to 0.21)<br>0.22(-0.28 to 0.72)<br>0.08(-0.41 to 0.58)<br>-2.18(-3.13 to -1.23)*<br>0.17(-0.32 to 0.67)	Pelv Obl Max Sw<br>Hip Abd Max Sw	0.13(-0.36 to 0.63)<br>-0.34(-0.85 to 0.17)	Hip Toe-Off
Nikamp, 2018	DelayedAFO / With AFO	Hip Max Sw<br>Knee Max Sw<br>Ankle Max Sw<br>Hip Toe-Off<br>Knee Toe-Off	-0.17(-0.72 to 0.38)<br>0.08(-0.47 to 0.63)<br>-0.10(-0.65 to 0.45)<br>-0.51(-1.09 to 0.08)<br>-0.04(-0.59 to 0.50)	Pelv Obl Max Sw<br>Hip Abd Max Sw	-0.26(-0.82 to 0.29)<br>-0.48(-1.06 to 0.10)	
Mahtani, 2017	Stepping Training / Experimental	Hip Max Sw<br>Knee Max Sw<br>Ankle Max Sw	0.40(-0.13 to 0.93)<br>0.00(-0.51 to 0.51)<br>0.19(-0.32 to 0.71)	Hip Abd Max Cycle<br>Ankle Lateral Disp Max Cycle	0.76(0.17 to 1.35)*<br>1.00(0.36 to 1.64)*	HipAbd Max Cycle<br>AnkleLateralDisp Max Cycle
Mahtani, 2017	Stepping Training / Pilot	Hip Max Sw<br>Knee Max Sw<br>Ankle Max Sw	0.56(-0.06 to 1.17)<br>0.49(-0.12 to 1.09)<br>0.41(-0.19 to 1.00)	Hip Abd Max Cycle<br>Ankle Lateral Disp Max Cycle	0.78(0.12 to 1.44)*<br>0.53(-0.08 to 1.14)	HipAbd Max Cycle
Mahtani, 2017	Stepping Training / Control	Hip Max Sw<br>Knee Max Sw<br>Ankle Max Sw	0.18(-0.33 to 0.69)<br>-0.07(-0.58 to 0.44)<br>-0.06(-0.57 to 0.45)	Hip Abd Max Cycle<br>Ankle Lateral Disp Max Cycle	-0.06(-0.57 to 0.45)<br>0.28(-0.24 to 0.81)	
Qian, 2015	FES Training TARF / Experimental	Knee Max Cycle	0.60(-0.08 to 1.29)	Foot Lateral Disp Max Cycle	-1.09(-1.90 to -0.27)*	FootLateralDisp Max Cycle
Shin, 2015	RAS Training / Experimental	Hip Max Sw<br>Knee Max Sw<br>Hip Toe-Off	0.00(-0.44 to 0.44)<br>0.26(-0.19 to 0.71)<br>-0.15(-0.59 to 0.30)	Hip Abd Mid Sw<br>Hip ER Max Sw	0.22(-0.23 to 0.66)<br>-0.15(-0.60 to 0.29)	
Wang, 2018	Acupuncture / Experimental group	Hip Max Cycle<br>Knee Max Cycle	-0.38(-0.87 to 0.12)<br>0.19(-0.29 to 0.67)	Hip Abd Max Cycle	-0.02(-0.50 to 0.46)	
Wang, 2018	Acupuncture / Control group	Hip Max Cycle<br>Knee Max Cycle	-0.32(-0.81 to 0.17)<br>0.34(-0.16 to 0.83)	Hip Abd Max Cycle	0.60(0.07 to 1.12)*	HipAbd Max Cycle
Dumont-Casalechi, 2022	Sham stimulation	Knee Max Sw<br>Ankle Max Sw	0.15(-0.42 to 0.72)<br>0.21(-0.36 to 0.78)	Hip Abd Max Cycle	0.38(-0.21 to 0.97)	
Dumont-Casalechi, 2022	10J stimulation	Knee Max Sw<br>Ankle Max Sw	0.05(-0.52 to 0.61)<br>0.21(-0.37 to 0.78)	Hip Abd Max Cycle	0.62(-0.01 to 1.24)	
Dumont-Casalechi, 2022	30J stimulation	Knee Max Sw<br>Ankle Max Sw	0.01(-0.55 to 0.58)<br>0.04(-0.53 to 0.61)	Hip Abd Max Cycle	0.56(-0.05 to 1.18)	
Dumont-Casalechi, 2022	50J stimulation	Knee Max Sw<br>Ankle Max Sw	0.07(-0.50 to 0.64)<br>0.11(-0.46 to 0.68)	Hip Abd Max Cycle	0.37(-0.22 to 0.95)	
		Knee Max Sw	1.03(0.41 to 1.66)*	Hip Abd Max Cycle	0.09(-0.40 to 0.59)	Knee Max Sw

Dumont-Cimolin, 2022	1 TDCS / Experimental group	Ankle Max Sw	0.57(0.03 to 1.11)*			Ankle Max Sw
Dumont-Cimolin, 2022	10 TDCS / Experimental group	Knee Max Sw<br>Ankle Max Sw	1.68(0.88 to 2.47)*<br>0.90(0.31 to 1.50)*	Hip Abd Max Cycle	0.07(-0.43 to 0.56)	Knee Max Sw<br>Ankle Max Sw
Dumont-Cimolin, 2022	1 month follow up / Experimental group	Knee Max Sw<br>Ankle Max Sw	2.14(1.21 to 3.08)*<br>0.80(0.23 to 1.38)*	Hip Abd Max Cycle	0.16(-0.34 to 0.65)	Knee Max Sw<br>Ankle Max Sw
Dumont-Cimolin, 2022	1 TDCS / Control group	Knee Max Sw<br>Ankle Max Sw	-0.04(-0.53 to 0.45)<br>0.37(-0.15 to 0.88)	Hip Abd Max Cycle	0.18(-0.32 to 0.67)	
Dumont-Cimolin, 2022	10 TDCS / Control group	Knee Max Sw<br>Ankle Max Sw	-0.08(-0.58 to 0.41)<br>0.29(-0.22 to 0.79)	Hip Abd Max Cycle	0.23(-0.27 to 0.74)	
Dumont-Cimolin, 2022	1 month follow up / Control group	Knee Max Sw<br>Ankle Max Sw	0.04(-0.45 to 0.53)<br>0.24(-0.26 to 0.74)	Hip Abd Max Cycle	0.32(-0.19 to 0.83)	
**Without and with orthoses comparison**						
Nikamp, 2018	Early AFO / PRE	Hip Max Sw<br>Knee Max Sw<br>Ankle Max Sw<br>Hip Toe-Off<br>Knee Toe-Off	0.04(-0.45 to 0.53)<br>-0.11(-0.60 to 0.39)<br>0.70(0.14 to 1.25)*<br>-0.02(-0.51 to 0.47)<br>-0.06(-0.56 to 0.43)	Pelv Obl Max Sw<br>Hip Abd Max Sw	-0.11(-0.60 to 0.39)<br>-0.04(-0.53 to 0.46)	Ankle Max Sw
Nikamp, 2018	Delayed AFO / PRE	Hip Max Sw<br>Knee Max Sw<br>Ankle Max Sw<br>Hip Toe-Off<br>Knee Toe-Off	0.01(-0.54 to 0.55)<br>0.05(-0.50 to 0.59)<br>0.50(-0.08 to 1.08)<br>0.03(-0.52 to 0.57)<br>0.12(-0.43 to 0.66)	PelvObl Max Sw<br>HipAbd Max Sw	-0.20(-0.76 to 0.35)<br>0.00(-0.55 to 0.55)	
Nikamp, 2018	Early AFO / POST	Hip Max Sw<br>Knee Max Sw<br>Ankle Max Sw<br>Hip Toe-Off<br>Knee Toe-Off	0.12(-0.36 to 0.60)<br>-0.01(-0.49 to 0.47)<br>0.25(-0.24 to 0.73)<br>-1.18(-1.82 to -0.54)*<br>-0.01(-0.49 to 0.47)	PelvObl Max Sw<br>HipAbd Max Sw	-0.13(-0.61 to 0.35)<br>-0.03(-0.50 to 0.45)	Hip Toe-Off
Nikamp, 2018	Delayed AFO / POST	Hip Max Sw<br>Knee Max Sw<br>Ankle Max Sw<br>Hip Toe-Off<br>Knee Toe-Off	0.07(-0.47 to 0.62)<br>0.00(-0.54 to 0.55)<br>0.60(0.00 to 1.20)*<br>-0.16(-0.71 to 0.39)<br>-0.01(-0.55 to 0.54)	Pelv Obl Max Sw<br>Hip Abd Max Sw	-0.12(-0.67 to 0.42)<br>0.10(-0.45 to 0.65)	Ankle Max Sw
Awad, 2017	Unilateral Exo Suit / Experimental	Knee Max Sw<br>Ankle Max Sw	0.15(-0.47 to 0.77)<br>0.77(0.05 to 1.49)*	Hip Hiking Max Sw<br>Foot Lateral Disp Max Sw	-0.40(-1.05 to 0.24)<br>-0.43(-1.08 to 0.22)	
Nikamp, 2017	AFO / Experimental	Hip Max Sw<br>Knee Max Sw<br>Ankle Max Sw<br>Hip Toe-Off<br>Knee Toe-Off	0.03(-0.39 to 0.45)<br>0.02(-0.40 to 0.45)<br>0.64(0.17 to 1.10)*<br>0.00(-0.42 to 0.42)<br>0.12(-0.30 to 0.55)	Pelv Obl Max Sw	-0.17(-0.60 to 0.25)	Ankle Max Sw
Boudarham, 2014	AFO Liberte / Experimental	Ankle Max Sw	0.68(0.08 to 1.27)*	Pelv Obl Max Sw	-0.14(-0.67 to 0.39)	Ankle Max Sw
Cruz, 2009	AFO / Experimental	Hip Max Sw<br>Knee Max Sw<br>Ankle Max Sw<br>Hip Toe-Off<br>Knee Toe-Off<br>Toe Clear Min Sw	0.14(-0.46 to 0.73)<br>-0.33(-0.94 to 0.28)<br>1.04(0.28 to 1.81)*<br>-0.13(-0.73 to 0.46)<br>-0.37(-0.98 to 0.25)<br>0.00(-0.59 to 0.59)	Pelv Obl Max Sw<br>Hip Abd Max Sw	-0.61(-1.27 to 0.04)<br>0.23(-0.37 to 0.82)	Ankle Max Sw
Zollo, 2015	Solid AFO / Experimental	Ankle ROM Sw<br>Ankle Max Sw<br>Knee Max Sw<br>Hip ROM Sw<br>Hip Max Sw	-0.37(-0.96 to 0.22)<br>0.52(-0.09 to 1.13)<br>-0.28(-0.86 to 0.30)<br>0.34(-0.25 to 0.92)<br>0.08(-0.49 to 0.64)	Hip Abd ROM Sw<br>Pelv Obl ROM Sw	-0.19(-0.76 to 0.39)<br>-0.02(-0.59 to 0.55)	
Zollo, 2015	Dynamic AFO / Experimental	Ankle ROM Sw<br>Ankle Max Sw<br>Knee Max Sw<br>Hip ROM Sw<br>Hip Max Sw	-0.49(-1.09 to 0.12)<br>0.15(-0.42 to 0.72)<br>-0.80(-1.47 to -0.13)*<br>0.04(-0.53 to 0.61)<br>-0.13(-0.70 to 0.44)	Hip Abd ROM Sw<br>Pelv Obl ROM Sw	-0.31(-0.90 to 0.27)<br>-0.12(-0.69 to 0.45)	Knee Max Sw

Abd: abduction; AFO: Ankle-foot orthosis; Clear: clearance; Disp: displacement; ER: external rotation; FES: functionnal electrical stimulation; Max: maximal; Min: minimal; Obl: obliquity; Pelv: pelvis; ROM: Range of motion; RAS: gait training with rythmic auditory system; Sw: Swing; TARF: Training with FES during 4 weeks on the Tibialis anterior and the Rectus femoris.
